# Boosting Photocatalytic CO_2_ Cycloaddition via Dual-Active Site Coordination over Amino-Functionalized UiO-66(Zr)

**DOI:** 10.3390/molecules31050902

**Published:** 2026-03-09

**Authors:** Yajing Lv, Haohao Yan, Wenhui Ye, Lin Ye, Jinmei Chen, Yutong Lin, Shuying Zhu, Dengrong Sun, Xiyao Liu, Ruowen Liang

**Affiliations:** 1 College of Chemistry, Fuzhou University, Fuzhou 350002, China; lyj010423@163.com (Y.L.); y15038159528@163.com (H.Y.); yyewenhui@163.com (W.Y.); syzhu@fzu.edu.cn (S.Z.); 2 College of Carbon Neutrality Future Technology, Sichuan University, Chengdu 610064, China; yelin312@scu.edu.cn; 3 Fujian Provincial Key Laboratory of Featured Biochemical and Chemical Materials, Ningde Normal University, Ningde 352100, China; 15160303767@163.com (J.C.); 13489059799@163.com (Y.L.); 4 Fujian Province University Key Laboratory of Green Energy and Environment Catalysis, Ningde Normal University, Ningde 352100, China

**Keywords:** carbon dioxide fixation, epoxide cycloaddition, metal–organic frameworks, dual-active sites, visible-light photocatalysis, UiO-66(Zr)-NH_2_

## Abstract

CO_2_ cycloaddition with epoxides offers a sustainable route for CO_2_ utilization, yet the simultaneous activation of both substrates remains challenging. Herein, using UiO-66(Zr)-NH_2_ (denoted as UZN) as a model system, we illustrate that dual-active sites consisting of unsaturated Zr^4+^ centers and amine groups can efficiently accelerate CO_2_ fixation with epoxides under visible light. The unique ensemble in UZN optimizes light harvesting, promotes charge carrier separation, and enriches bifunctional active sites for efficient adsorption and activation of epoxides and CO_2_. Consequently, UZN exhibits significantly improved CO_2_-epoxide cycloaddition performance compared to UiO-66(Zr)-H (denoted as UZH), achieving a PC yield of 99.5%, with a production rate of 9.97 mmol·g^−1^·h^−1^. This work establishes a clear coordination–photocatalytic synergy in MOF-based systems and provides fundamental insights and a generalizable strategy for designing advanced catalysts for CO_2_ transformation.

## 1. Introduction

Global industrialization and rapid urban expansion have led to excessive fossil fuel consumption, accelerating both global warming and ocean acidification. Among various carbon-mitigation strategies, the chemical conversion of CO_2_ into high-value chemicals has been considered an effective means for CO_2_ resource utilization. Chemical fixation of CO_2_ with epoxides to produce cyclic carbonates is an ideal pathway due to its 100% atom economy. The resulting cyclic carbonates, which serve as lithium battery electrolytes, low-toxicity solvents, and pharmaceutical intermediates, have therefore attracted widespread attention.

Mechanistically, the ring-opening of the epoxide, typically initiated by nucleophilic attack of a halide anion, is often regarded as the rate-determining step (RDS). However, the activation of CO_2_ is equally essential to the overall catalytic efficiency. Owing to its linear geometry, high C=O bond energy (+805 kJ·mol^−1^) and thermodynamic inertness, CO_2_ activation requires substantial input energy, thereby limiting reaction efficiency under conventional thermal conditions. In recent years, photocatalysis has emerged as an appealing strategy to address this challenge because photogenerated electrons and holes can activate small molecules, such as CO_2_, NO, N_2_O, CH_4_, and benzyl alcohols, by lowering kinetic barriers and enabling energetically challenging transformations under mild conditions [1,2,3,4]. These photophysical advantages make photocatalysis an ideal approach for promoting CO_2_^−^ epoxide cycloaddition reactions in an energy- and cost-effective manner. To date, various catalysts featuring Lewis acidic sites (e.g., Zn-loaded porous carbon, Al-loaded porous carbon and W_18_O_49_/g-C_3_N_4_) have been designed and demonstrated appreciable catalytic performance toward the activation of individual reactants [5,6,7]. Nevertheless, most of these systems focus on activating either CO_2_ or epoxides alone, while studies explicitly addressing the synergistic activation of both substrates via Lewis acid–base cooperation remain scarce.

Metal–organic frameworks (MOFs), owing to their multifunctional sites and architectural versatility, have emerged as promising catalysts for chemically converting CO_2_ into cyclic carbonates [8,9]. Especially UiO-66-NH_2_ (denoted as UZN), a representative Zr-based MOF constructed from hexanuclear Zr_6_O_4_(OH)_4_ secondary building units connected by 2-aminoterephthalate (ATA) linkers, has been widely reported as a visible-light-responsive photocatalyst for CO_2_ reduction and selective organic transformations. More importantly, the Zr_6_ nodes in UZN can generate coordinatively unsaturated Zr^3+^ sites upon defect formation, which function as strong Lewis acidic centers for CO_2_ adsorption and activation. Simultaneously, the -NH_2_ groups on the ATA linkers serve as intrinsic Lewis basic sites capable of interacting with epoxide substrates and facilitating charge localization under light irradiation.

Among various MOFs, UiO-66-NH_2_ (denoted as UZN), a representative Zr-based MOF constructed from hexanuclear Zr_6_O_4_(OH)_4_ secondary building units connected by 2-aminoterephthalate (ATA) linkers, stands out as a potential photocatalyst for the effective cycloaddition of CO_2_ owing to the following reasons: (1) Exposed Zr metal centers in UZN may act as Lewis acid sites for selective adsorption and coordination activation of CO_2_. The highly coordinated and compact inorganic secondary building units of UZN facilitate preferential CO_2_ uptake. (2) Meanwhile, the amine groups assist in epoxide polarization and act as hole accumulation sites for Br^−^ oxidation, thereby accelerating the epoxide ring-opening. It is noteworthy that a perylene-3,4,9,10-tetracarboxylic diimide-UiO-66-NH_2_ composite (denoted as PDI-UZN) was previously developed by our group [10], in which the built-in electric field between exposed Zr sites and PDI significantly enhanced catalytic performance by promoting charge separation and carrier dynamics. However, although CO_2_ adsorption on unsaturated Zr sites was demonstrated, a more detailed understanding of epoxide activation and the cooperative roles of dual-active sites under visible light remains to be explored. Therefore, a systematic investigation of the cooperative adsorption and activation roles of the dual-active sites in UZN is necessary.

Herein, UZN was synthesized and identified as an efficient photocatalyst for visible-light-driven cycloaddition of CO_2_ with epoxides. Combining in situ EPR and in situ infrared spectroscopy, the distinct roles of the dual-coordination sites are uncovered. The unsaturated Zr^4+^ nodes act as Lewis acid centers, selectively adsorbing CO_2_ and generating •CO_2_^−^ species via electron transfer. Meanwhile, the amino ligands promote epoxide adsorption, where the interaction is primarily governed by weak non-covalent interactions consistent with van der Waals forces. Density functional theory (DFT) calculations provide adsorption energies and reaction pathways consistent with the proposed dual-site interaction, contributing to a better understanding of the coordination-assisted catalytic process and providing deeper insight into the coordination activation–catalysis pathway.

## 2. Results

### 2.1. Characterizations

**Figure 1:**
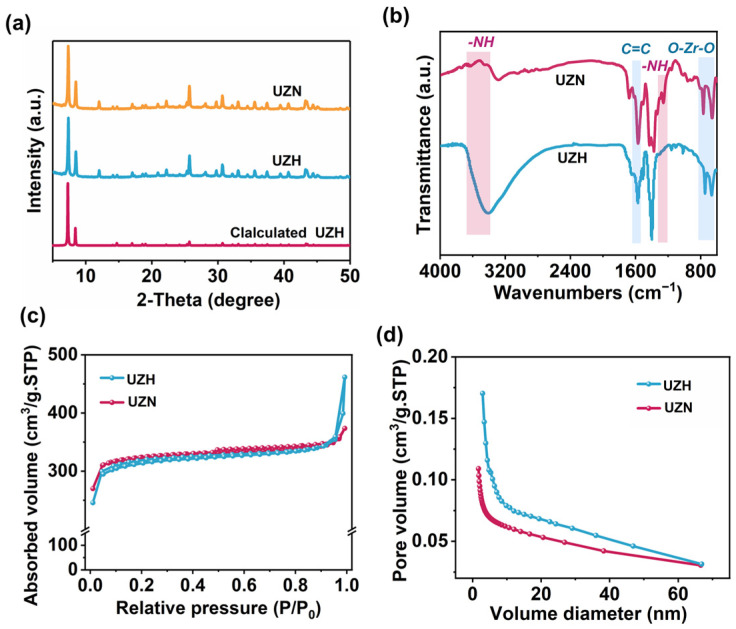
(**a**) XRD patterns, (**b**) FT-IR spectrum, (**c**) N_2_ adsorption–desorption isotherms of the samples, and (**d**) pore size distribution curves of the photocatalysts.

The X-ray diffraction (XRD) pattern of UZN shows well-defined peaks consistent with the simulated UiO-66(Zr) structure (Figure 1a), indicating its high crystallinity. The nearly identical diffraction profiles of UZN and UZH further indicate that introducing -NH_2_ groups does not alter the framework topology [11,12,13]. FTIR spectra (Figure 1b) also verify the successful incorporation of -NH_2_ functionalities [14,15,16]. Specifically, the symmetric and asymmetric N-H vibrations at 3623 and 3423 cm^−1^ are characteristic of NH_2_ groups. The band at 1265 cm^−1^ can be ascribed to the stretching vibration of N-C, while the peak at 1578 cm^−1^ is attributed to the stretching of the C=C bond in the aromatic ring, and the strong bands at 769 and 658 cm^−1^ are attributed to the O-Zr-O bonds, further supporting the successful synthesis of UZN. In addition, scanning electron microscopy (SEM), transmission electron microscopic (TEM), nitrogen adsorption, and XPS analyses collectively support the successful synthesis of UZN with the expected morphology, porosity, and surface chemical composition (Figures S1–S3).

**Figure 2:**
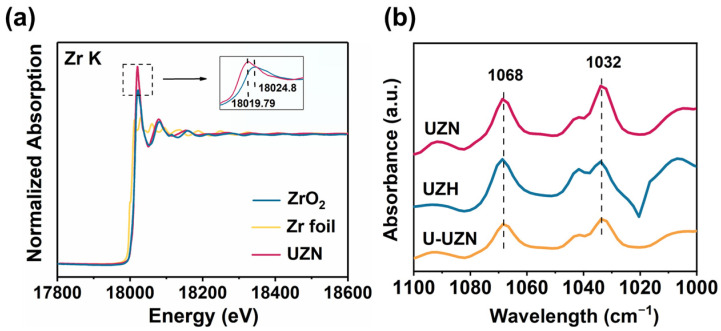
(**a**) XANES spectra for UZN, (**b**) Py-FTIR spectra for UZH, UZN, and U-UZN.

The coordination environment of Zr centers in UZN was investigated by X-ray absorption near-edge structure (XANES) spectroscopy using Zr foil and ZrO_2_ as references. As shown in Figure 2a, the absorption edge position of UZN, determined from the maximum of the first derivative of the XANES spectrum, is located at 18,013.9 eV, which lies between that of Zr foil (17,977.8 eV) and ZrO_2_ (18,019.0 eV), and is closer to that of ZrO_2_. This result indicates that Zr in UZN predominantly exists in a high-valence state, with possible contributions from a mixed Zr^3+^/Zr^4+^ environment. The intense white line feature observed in the inset of Figure 2a can be assigned to the dipole-allowed 1 s → 5 p transition, which is consistent with previous reports on Zr-based carboxylate MOFs [17,18]. The absorption edge and intensity of the white line in UZN (18,019.9 eV) are relatively lower than those of ZrO_2_ (18,020.2 eV), suggesting a loss of ligands in the first coordination shell around Zr [18,19,20]. Fitting of the Fourier-transformed extended X-ray absorption fine structure (FT-EXAFS) data may provide insight into the structural parameters such as coordination number (CN), bond distance, and local disorder (Debye–Waller factor) (Table S1). As can be seen, UZN mainly contains first-shell Zr-O coordination and second-shell Zr-Zr coordination, in which the total coordination number of Zr-O in the first shell is approximately 6.6, lower than the full coordination number of 8. This is consistent with the unsaturated coordinate structure of Zr. The unsaturated sites provide abundant active sites for the adsorption and activation of reactants. Utilizing probe molecules, such as pyridine or ammonia, in combination with spectroscopic techniques has become a well-established methodology to titrate active sites [19,21,22]. To experimentally probe the Lewis acidity associated with the unsaturated Zr sites, pyridine adsorption Fourier-transform infrared spectroscopy (Py-FTIR) was employed (Figure 2b). Although the characteristic pyridine bands associated with Lewis acid sites typically appear in the 1400–1600 cm^−1^ region, these signals are often masked in MOF materials due to strong carboxylate and aromatic ring vibrations [23,24,25,26]. According to previous work, signal peaks in the range of 1000–1100 cm^−1^ can also be used as a reference [27]. The intensity of Lewis acid strength in the three samples can be determined from two major signal peaks at 1032 and 1068 cm^−1^, which can be assigned to pyridine coordinated to Zr sites. To verify this conjecture, controlled experiments were conducted, and unactivated UZN (U-UZN, meaning it contains a less unsaturated metal center) was adopted as the contrast sample. As shown in Figure 2b and Table S2, UZN exhibits significantly stronger absorption bands at 1032 and 1068 cm^−1^ compared with U-UZN, indicating a higher density of accessible Lewis acidic Zr sites. These results are in good agreement with the EXAFS analysis and collectively confirm the presence of abundant unsaturated Zr coordination sites in UZN.

**Figure 3:**
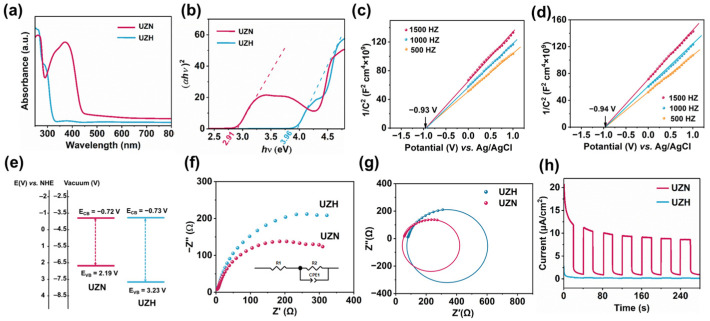
(**a**) UV-vis DRS spectra and (**b**) Tauc plots of the resulting samples; (**c**) Mott–Schottky plot of UZH; (**d**) Mott–Schottky plot of UZN; (**e**) band structures of UZH and UZN; (**f**) electrochemical impedance spectroscopy (EIS) Nyquist plots of the samples, the inset shows the equivalent circuit diagram; (**g**) fitted circle from the EIS Nyquist plots; and (**h**) transient photocurrent representations of UZH and UZN.

As shown in Figure 3a,b, UZN exhibits an optical band gap (E_g_) of 2.91 eV with a pronounced absorption edge extending into the visible-light region, indicating its strong potential for solar energy utilization. To further elucidate the electronic band structure, the flat-band potentials of UZN and UZH were estimated from the Mott–Schottky plots (Figure 3c,d). Both samples display positive slopes, characteristic of n-type semiconductor behavior. The flat-band potentials were determined to be approximately −0.93 V and −0.94 V vs. Ag/AgCl (pH = 7) for UZN and UZH, respectively. The potentials measured versus Ag/AgCl were converted to the NHE scale according to E_NHE_ = E_Ag/AgCl_ + 0.21 V (298 K). Since both samples exhibit typical n-type semiconductor behavior, the flat-band potentials are approximately equal to the conduction band minimum. Accordingly, the CB positions of UZN and UZH were estimated to be −0.72 V and −0.73 V vs. NHE, respectively. Based on the relationship E_VB-NHE_ = E_CB-NHE_ + E_g_, the valence band (VB) positions of UZN and UZH were calculated to be 2.19 and 3.23 eV, respectively (Figure 3e). This indicates that the introduction of the amino group reduced the redox potential of the VB of UZN. As a result, the photoabsorption edge of UZN shifts to the visible-light region.

Electrochemical impedance spectra (EIS) investigation was used to reveal the electrical conductivity of the samples [28,29]. The impedance data were fitted with Z-view 2.6.0.11 software to draw an equivalent circuit model [30,31]. Figure 3f shows the electrochemical impedance spectroscopy (EIS) results, which reflect that UZN shows a smaller impedance arc radius (Figure 3g), proving excellent photoexcited carrier transport and separation in UZN. A similar result can also be found in the photoelectric response tests, which were recorded over several on–off cycles of intermittent irradiation. Furthermore, as shown in Figure 3h, a stable and strong short-circuit photocurrent response has been observed for the UZN photoelectrode under visible-light irradiation. However, no obvious circuit photocurrent has been detected for UZH, which is consistent with the UV-vis DRS results.

### 2.2. Photocatalytic CO_2_ Fixation with Epoxides

**Figure 4:**
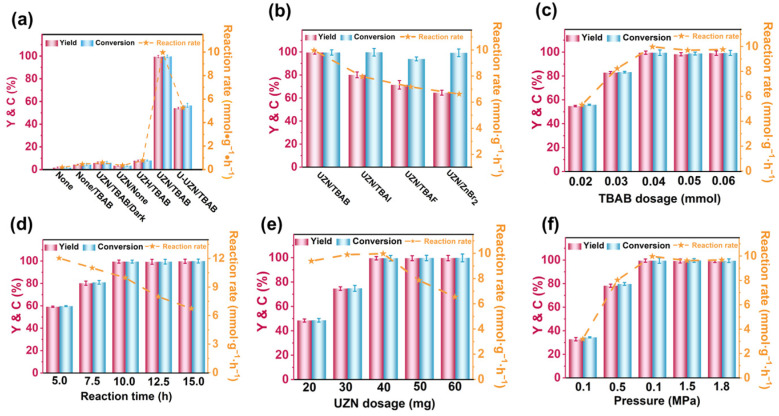
Photocatalytic CO_2_ fixation with PO over UZN under various conditions: (**a**) blank experiments, (**b**) different co-catalysts, (**c**) co-catalyst dosage, (**d**) reaction time, (**e**) photocatalyst dosage, and (**f**) CO_2_ pressure. Reaction conditions: solvent CH_3_CN (16 mL), visible light (100 mW·cm^−2^, λ ≥ 420 nm). The product yields were quantified by GC-FID with CFCl_3_ as an internal standard.

The photocatalytic activities samples were evaluated via CO_2_ cycloaddition with PO under visible-light irradiation. As shown in Figure 4a (Table S3, Entry 1–2), negligible PC formation was observed in the absence of the photocatalyst, indicating that the catalyst is essential for the reaction. Furthermore, a control experiment was performed under identical temperature (300 K) conditions in the dark (Table S3, Entry 3). Under dark conditions, the reaction rate was only 0.59 mmol·g^−1^·h^−1^, which is approximately 17 times lower than that under visible-light irradiation (9.97 mmol·g^−1^·h^−1^). This pronounced difference clearly demonstrates that light irradiation plays a decisive role in promoting the reaction, confirming the photocatalytic nature of the system. In addition, when TBAB was excluded from the reaction system, only a trace amount of PC was produced (3.5% yield, Table S3, Entry 4), highlighting the essential role of the co-catalyst. Upon introducing TBAB, UZN exhibited remarkable photocatalytic performance, achieving a PC yield of 99.5% with a reaction rate of 9.97 mmol·g^−1^·h^−1^ after 10 h of visible-light irradiation (λ ≥ 420 nm) (Table S3, Entry 6). In contrast, pristine UZH delivered much lower activity under identical conditions (Table S3, Entry 5). The superior performance of UZN can be attributed to its enhanced visible-light absorption and the coordination activation capability arising from unsaturated Zr^4+^ sites. To further identify the nature of the active sites, controlled experiments were conducted using an unactivated UZN sample (U-UZN), which contains fewer coordination-unsaturated Zr centers. As shown in Table S3 (Entry 7), U-UZN exhibits a significantly reduced reaction rate (5.26 mmol·g^−1^·h^−1^), confirming that the exposed Zr^4+^ centers play a critical role as Lewis acidic active sites for CO_2_ activation during the photocatalytic cycloaddition process [32]. The apparent quantum yield (AQY) was determined at different wavelengths, and a benchmark AQY of 15.1% was obtained at 350 nm. The wavelength-dependent AQY trend closely follows the DRS absorption profile of UZN. These results indicate that the reaction rate correlates with photon absorption, supporting the photon-driven nature of the process.

As shown in Figure S4a,b, GC-MS analysis of the product exhibits a characteristic fragment peak at *m*/*z* = 87.1, corresponding to the -C_3_H_3_O_3_ fragment of PC. Moreover, the ^13^C NMR spectrum (Figure S5) further confirms the formation of PC. The involvement of CO_2_ in the cycloaddition reaction was further verified by isotopic labeling experiments using ^13^CO_2_ (Figure S4c,d). As expected, when ^13^CO_2_ was introduced, the *m*/*z* value of the target product changes to 88.1 (-^13^CC_2_H_3_O_3_), confirming the direct incorporation of CO_2_ into the carbonate product. Furthermore, no carbonate species were detected when CO_2_ was replaced by Ar (Figure S6), unambiguously demonstrating that all cyclic carbonate products originate from CO_2_.

Co-catalyst effects were also investigated in this series of reactions, and it was determined that the PC yield decreased in the order tetrabutylammonium bromide (TBAB) > tetrabutylammonium iodide (TBAI) > tetrabutylammonium fluoride (TBAF) > zinc bromide (ZnBr_2_) (Figure 4b and Table S4). This trend can be rationalized by the combined influence of nucleophilic strength and leaving-group ability of the co-catalysts, which directly affect the epoxide ring-opening step. Among them, TBAB exhibits the most favorable balance, highlighting a pronounced synergistic effect between TBAB and UZN in promoting CO_2_ cycloaddition.

The influence of reaction parameters, including catalyst dosage, TBAB dosage, reaction time, and CO_2_ pressure, was further examined (Figure 4c,f and Table S6). In all cases, volcano-shaped trends were observed, indicating that excessive amounts of catalyst or co-catalyst, or prolonged reaction times, lead to decreased activity. This phenomenon is likely attributed to partial blockage or occupation of active sites by carbonate species, resulting in reduced accessibility of the Lewis acidic Zr sites and gradual catalyst deactivation.

To clarify this discrepancy, the catalyst subjected to different light wavelengths was systematically characterized. XRD patterns show that although the crystal structure remains identifiable after UV exposure, the diffraction intensity decreases markedly, suggesting partial structural degradation (Figure S8). SEM and TEM images further reveal pronounced morphology distortion under UV irradiation, including local lattice collapse and particle fusion (Figures S9 and S10). This instability is likely associated with ligand photolysis or photo-oxidation of the Zr-oxo nodes, resulting in reduced active site density and accelerated charge carrier recombination [33,34,35]. These results collectively indicate that while UZN is photoactive across the UV-visible region, excessively high-energy UV light induces structural damage, thereby suppressing its intrinsic photocatalytic activity.

**Figure 5:**
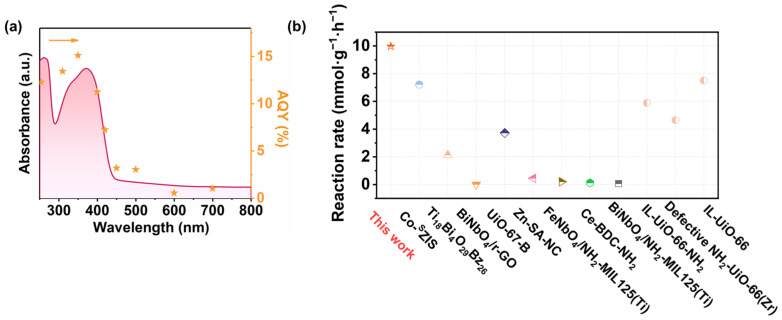
(**a**) AQY of the catalyst under different monochromatic light conditions, (**b**) comparisons of recent studies on photocatalytic CO_2_ fixation to produce PC.

**Figure 6:**
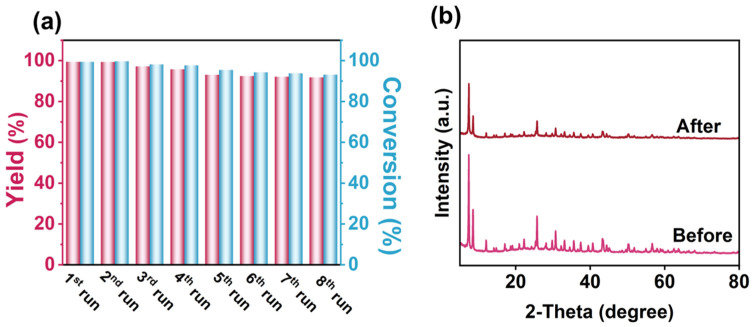
(**a**) Cycled experimental results and (**b**) XRD patterns of UZN before and after the reaction.

Owing to the excellent photocatalytic performance of CO_2_ fixation with PO, a variety of epoxides bearing different substituents were evaluated under identical reaction conditions. The products were confirmed by GC-MS (Figures S11 and S12). Taking a view of the results summarized in Table 1, high yields were obtained for less sterically hindered epoxides, such as those substituted with -Cl, -CH_2_CH_3_ and -(CH_3_)_2_), likely due to the higher electrophilicity of the epoxide carbon. In contrast, epoxides bearing long-chain alkyl substituents exhibited lower yields (17–18%) because steric hindrance slowed the nucleophilic ring-opening step. For comparison, Figure 5b and Table S8 summarize the performance of photocatalysts reported to date for PC generation. To the best of our knowledge, UZN ranks among the most active organic semiconductor-based photocatalysts, outperforming several previously reported systems, such as UiO-67-B (reaction rate: 3.72 mmol·g^−1^·h^−1^), Ce-BDC-NH_2_ (reaction rate: 0.12 mmol·g^−1^·h^−1^), and BiNbO_4_/NH_2_-MIL125(Ti) (reaction rate: 0.062 mmol·g^−1^·h^−1^) [6,36,37]. In addition, UZN exhibits good catalytic stability, retaining over 90% of its initial activity after six consecutive reaction cycles (Figure 6a). XRD, BET, and TEM analyses further support that the crystalline structure, porosity, and morphology of UZN are largely maintained after eight catalytic cycles, with XRD patterns showing no significant peak shifts, BET surface area decreasing by less than 10%, and TEM images indicating preserved particle morphology. (Figure 6b and Figure S13), demonstrating its structural robustness under similar photocatalytic reaction conditions.

### 2.3. Theoretical Study of CO_2_ Cycloaddition over UZN

**Figure 7:**
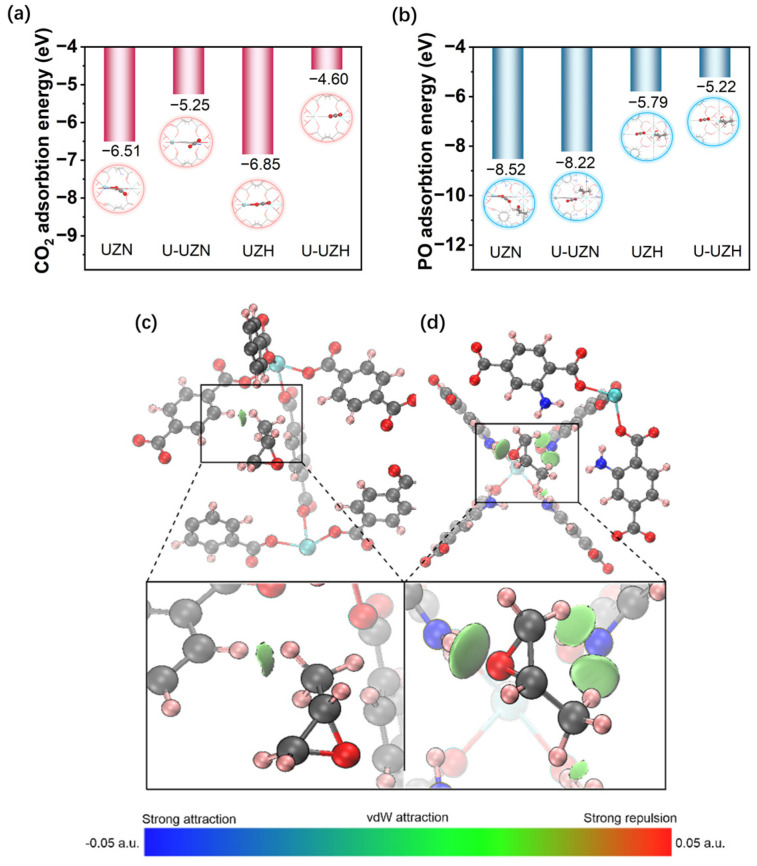
Calculated adsorption sites and adsorption energies of (**a**) CO_2_ and (**b**) PO on various catalyst models. IGMH analysis of (**c**) UZH and (**d**) UZN around PO (isovalue = 0.02 a.u.). Green regions indicate weak non-covalent interactions consistent with van der Waals forces (weaker than a standard hydrogen bond), while the cyan regions denote a more substantial level of attraction, albeit still less intense than conventional hydrogen bonds.

To shed light on the origin of the excellent performance of UZN in photocatalytic CO_2_ cycloaddition, density functional theory (DFT) calculations were performed. The adsorption energies of isolated CO_2_ and PO on UZN, U-UZN, UZH, and U-UZH were calculated, as shown in Figure 7a,b. The calculated CO_2_ adsorption energies on UZN and U-UZN are −6.51 and −5.25 eV, respectively, indicating a stronger interaction between CO_2_ and the exposed Zr sites in UZN. These results suggest that Lewis acidic Zr^4+^ centers may facilitate CO_2_ activation through enhanced adsorption. Upon removal of amino groups, both UZH and U-UZH exhibit substantially weaker CO_2_ adsorption, further underscoring the crucial role of exposed zirconium sites in CO_2_ activation. In contrast, PO adsorption is more strongly affected by the presence of amino groups. The adsorption energies on UZN and U-UZN (−8.52 and −8.22 eV) are significantly lower than those on amino-free UZH and U-UZH (−5.79 and −5.22 eV), implying enhanced PO–surface interactions mediated by the -NH_2_ functionality.

To further elucidate the molecular origin of this enhanced PO binding, we employed the Independent Gradient Model based on Hirshfeld partitioning (IGMH) to analyze the real-space non-covalent interaction regions between PO and the catalyst surface [38,39,40]. Unlike traditional models based on atomic coordinates, IGMH leverages wavefunction-derived electron density and its gradient, enabling high-resolution visualization of weak intermolecular forces through isosurface mapping. As shown in Figure 7c, the unfunctionalized UZH exhibits only a single, faint interaction region with PO, indicating weak van der Waals contact. In contrast, upon amino functionalization, UZN presents three pronounced interaction lobes between PO and the -NH_2_ sites, revealing a synergistic stabilization effect (Figure 7d). These results are consistent with the adsorption energy calculations and support the role of amino functionalization in enhancing PO adsorption strength while also promoting its polarization, thereby facilitating subsequent ring-opening steps. This cooperative dual-site activation rationalizes the experimentally observed superior activity of UZN in photocatalytic cyclic carbonate formation.

**Figure 8:**
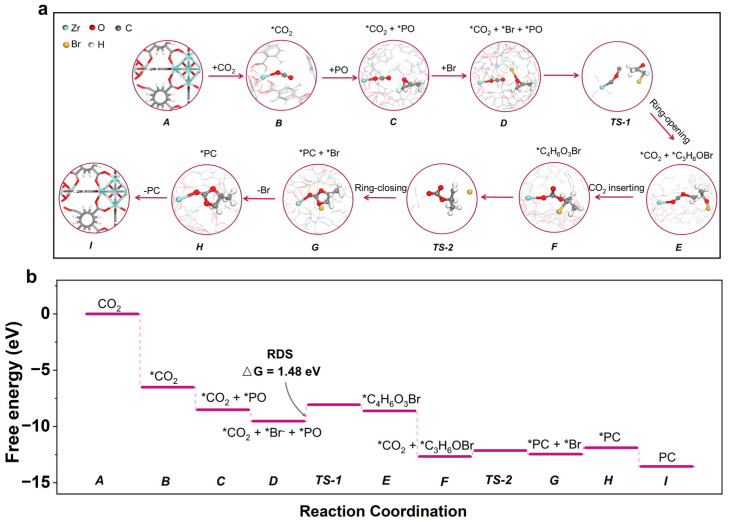
(**a**) Calculated configuration for CO_2_ cycloaddition and (**b**) potential energy diagrams of CO_2_ cycloaddition paths over UZN.

Having established how UZN activates both CO_2_ and PO, the complete reaction pathway of CO_2_ cycloaddition over UZN is illustrated in Figure 8a and Figure S15. By anchoring the oxygen atom of CO_2_, UZN (A) tends to form a Zr···O_CO2_ coordination state (B) between exposed Zr sites and CO_2_. PO is preadsorbed near the -NH_2_ groups through strengthened non-covalent interactions (C). Subsequently, Br is co-adsorbed to generate activated *Br (D), which participates in the ring-opening step to form the intermediate *C_3_H_6_OBr (E). Because this PO ring-opening step has the highest energy barrier among all steps, it is likely the rate-determining step under the calculated conditions for CO_2_ photofixation over UZN, consistent with the observed elongation and weakening of the C-O bond in PO. The ring-opened intermediate (C_3_H_6_OBr) is detected via GC-MS analysis when the reaction is operated in the absence of CO_2_ (Figure S14). After that, *CO_2_ is inserted into *C_3_H_6_OBr to form the alkyl carbonate intermediate *C_4_H_6_O_3_Br (F). *C_4_H_6_O_3_Br eventually undergoes a ring-closing step (G) to release Br (H) and PC (I).

### 2.4. Mechanistic Insights into Photocatalytic CO_2_ Fixation with Epoxides

**Figure 9:**
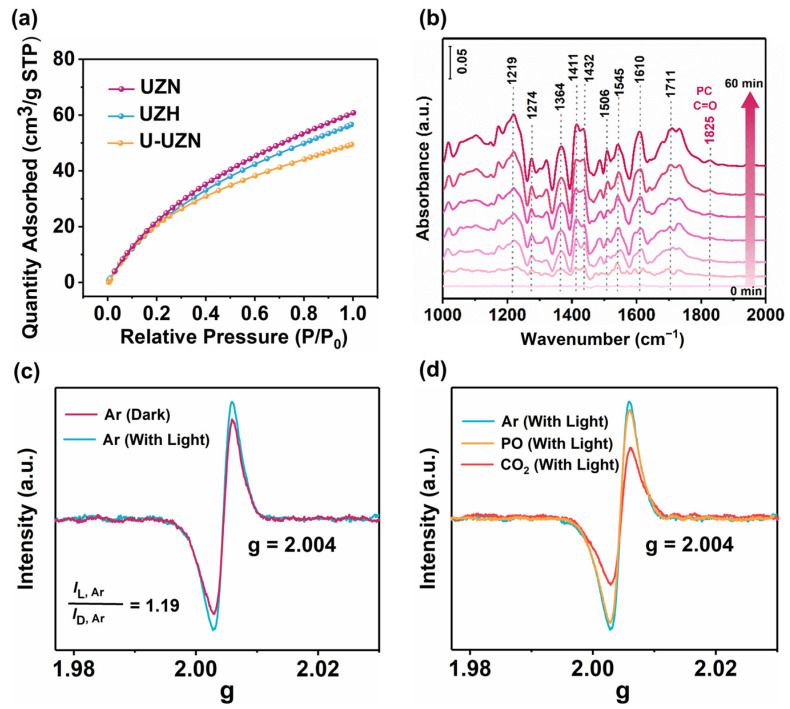
(**a**) CO_2_ adsorption isotherms at 298 K for UZN, UZH, and U-UZN; (**b**) in situ FTIR spectra of PC photosynthesis over UZN; (**c**) EPR spectra of UZN under CO_2_ in an Ar atmosphere; (**d**) EPR spectra of UZN under different atmospheres.

Since unique uncoordinated octahedral Zr^4+^ sites are observed in UZN, it is of interest to examine whether these sites provide unique advantages for the selective adsorption and activation of CO_2_ molecules. To address this, CO_2_ adsorption measurements were investigated, and the results are displayed in Figure 9a. Analysis of the CO_2_ adsorption isotherms shows that UZH exhibits a maximum CO_2_ uptake of 58.3 cm^3^/g, while UZN reaches 61.6 cm^3^/g, suggesting that the contribution of -NH_2_ groups to CO_2_ adsorption is minor. By contrast, the saturated coordination U-UZN sample exhibits a significantly reduced adsorption capacity of 44.5 cm^3^/g, supporting that exposed Zr^4+^ centers contribute to enhanced CO_2_ adsorption. This also ensures high-efficiency CO_2_ cycloaddition over UZN [41,42].

Time-dependent in situ DRIFT spectra was conducted to identify and dynamically monitor the surface-bound intermediate species during CO_2_ photoconversion on UZN. The detected surface species are labeled in Figure 9b. Absorption bands at 1219 and 1274 cm^−1^ are assigned to •CO_2_^−^ species, which serve as key intermediates for CO_2_ insertion to form the alkyl carbonate anion, and their intensities increase over time [10,43]. The significantly enhanced bands at around 1400–1800 cm^−1^ are ascribed to the contribution of the carbonate intermediate. In particular, the bands at 1506 and 1557 cm^−1^ are attributed to the twisting and scissoring vibrations of -CH_2_, respectively, while the bands appearing at 1610 and 1711 cm^−1^ belong to the C=O vibration of carbonate intermediates after CO_2_ insertion. Additional carbonate-related peaks at 1432 and 1364 cm^−1^ are assigned to the asymmetrical stretching vibration of O-C-O and the stretching vibration of C-O, due to the formation of R-OCOO on UZN. Furthermore, the product, i.e., PC, can be detected via C=O symmetric stretching at 1825 cm^−1^.

The in situ EPR spectra were employed to further recognize the reaction mechanism. Lorentzian lines with g values around 2.004 are attributed to Zr^3+^ in the 7%PDI-UZN. As evidenced in Figure S16, when H_2_ATA was irradiated with visible light, an ESR signal with a g value of 2.004 was observed, which can be attributed to spatially confined amino groups [44,45,46,47]. However, when UZN was irradiated, the above signal disappeared and a new ESR signal at g = 2.004 emerged. According to previous studies, the new signal can be ascribed to Zr^3+^, suggesting the ligand-to-metal charge-transfer (LMCT) process [48,49]. On the contrary, no ESR signal is observed over UZH under visible-light irradiation. As illustrated in Figure 9c, the ESR intensity of UZN in an inert argon atmosphere (I_L,Ar_) upon light irradiation is slightly higher than that in the dark condition (I_D,Ar_), suggesting effective charge separation of photogenerated electrons (*I*_L,Ar_/*I*_D,Ar_ = 1.19). In contrast, when CO_2_ is injected into the system, the intensity of the Zr^3+^ signal obviously declines (Figure 9d). The decrease in Zr^3+^ signal intensity upon CO_2_ introduction indicates that photoexcited electrons are efficiently transferred from UZN to CO_2_, which is consistent with the possible formation of •CO_2_^−^-derived radical intermediates and the regeneration of EPR-silent Zr^4+^. This finding is also congruent with the in situ FTIR results, where a considerable •CO_2_^−^ signal is detected. However, in a PO atmosphere, the intensity of the ESR signal was apparently higher than that in the CO_2_ atmosphere, suggesting that photogenerated electrons are not the primary active species responsible for PO ring-opening.

Radical-trapping experiments were conducted to probe the active species responsible for epoxide ring-opening. Traditionally, photogenerated holes are considered to polarize epoxides, enabling Br^−^ from nBu_4_NBr (TBAB) to attack and open the activated epoxide rings [50,51]. Considering the relatively low oxidation potential of TBAB in organic solvents (E_Br^−^/•Br_ = +0.92 V vs. NHE), oxidation of Br^−^ is thermodynamically more favorable than direct oxidation of the epoxide. To validate this, phenol was employed as a •Br scavenger (Figure S17), which significantly suppressed PC formation. To the best of our knowledge, the detection of the brominated product 4-bromophenol provides evidence consistent with the oxidation of Br- by photogenerated holes to generate •Br species (Figure S18), which participate in ring-opening of PO adsorbed on the -NH_2_ site. The PO ring-opening intermediate, C_3_H_6_OBr, is also obtained when the reaction is operated in the absence of CO_2_ (Figure S19). Importantly, the exclusive formation of cyclic carbonates and the detection of C_3_H_6_OBr, together with the absence of observable C-C bond cleavage products within analytical limits, suggest that a C-C fragmentation pathway of PO is unlikely under the present conditions. Moreover, no 4-bromophenol is detectable in dark, further supporting that visible-light irradiation is essential for radical generation (Figure S18).

**Figure 10:**
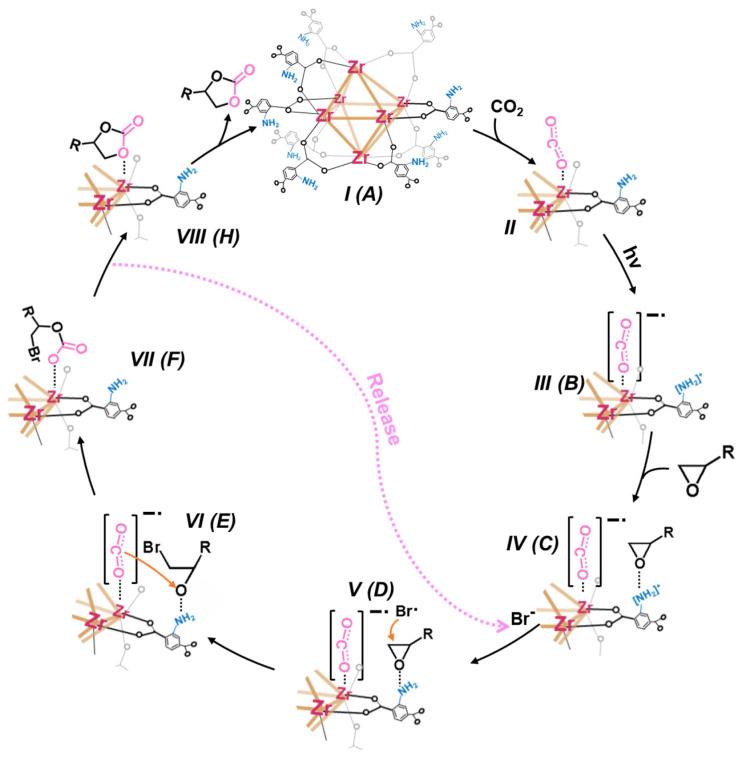
Mechanistic details of photocatalytic PC generation, where the alphabet in the brackets refer to the models in Figure 8. (The orange arrow indicates the chemical attack process and the brown line represents the Zr-Zr chemical bond).

Based on the above results, a possible photocatalytic pathway for CO_2_ fixation over UZN is proposed (Figure 10). Under visible-light irradiation, the -NH_2_ functional ligand acts as a light-harvesting antenna and initiates LMCT, producing electron–hole pairs. The photoexcited electrons migrate to the unsaturated Zr centers, where CO_2_ is adsorbed and activated via Lewis acid coordination. Although the CB potential (−0.72 V vs. NHE) is insufficient to reduce free linear CO_2_ in solution (E_CO_2_/•CO_2_^−^_ = −1.9 V vs. NHE), the coordination-induced activation of CO_2_ lowers the interfacial electron transfer barrier, enabling the formation of surface-bound •CO_2_^−^ species (steps II, III). Photogenerated holes localize on the amine sites and oxidize Br^−^ to •Br radicals (steps IV, V), which selectively attack the β-carbon of adsorbed PO, opening the ring. The reactive •CO_2_^−^ species subsequently insert into the ring-opened intermediate (step VI) to generate the CO_2_-PO adduct (step VII), which subsequently undergoes intramolecular ring closure to yield PC and regenerate Br^−^ for the next cycle (step VIII). Overall, the enhanced catalytic performance is associated with the synergistic coupling between Lewis acidic Zr sites for CO_2_ activation and Lewis basic -NH_2_ sites for hole accumulation and Br^−^ oxidation, wherein Lewis acid/base-mediated substrate activation cooperates with photoinduced electronic effects to enable efficient photocatalytic cycloaddition.

## 3. Materials and Methods

### 3.1. Preparation of UZN

UZN and UZH were prepared according to the reported studies [52,53], and detailed synthesis and characterization procedures can be found in the electronic Supporting Information. To further confirm the critical roles of the exposed Zr centers in coordination activation, unactivated UZN (UA-UZN, containing a less unsaturated metal center) was also prepared.

### 3.2. Evaluation of Photocatalytic Activity

A 100 mL high-pressure stainless-steel autoclave was used as the reactor for cycloaddition reaction, equipped with a magnetic stirrer and a temperature control system. A 300 W xenon lamp (PLS-SXE 300D, Beijing Perfectlight Co., Ltd., Beijing, China) with bandpass filters was used as the light source. A mixture of propylene epoxide (PO, 10 mmol), catalyst (10 mg), and 0.01 mmol co-catalyst (TBAB) was dissolved in acetonitrile (16 mL). The above mixture was transferred to the high-pressure stainless-steel autoclave, filled with molecular CO_2_ at a pressure of 1.0 MPa, and maintained for 10 h. After that, the reactor was cooled down to room temperature, and then the residual carbon dioxide pressure was gradually reduced. The catalysts were separated by filtration. The reaction mixtures were analyzed on a gas chromatograph (GC-2012, Shimazu, Tokyo, Japan) equipped with a capillary column and flame ionization detector (see the Supplementary data for details).

The yield of propylene carbonate (PC) and the selectivity for PC were defined as follows:

Conversion (%) = [(C_0_ − C_PO_)/C_0_] × 100 (1)

Yield (%) = C_PC_/C_0_ × 100 (2)

Selectivity (%) = [C_PC_/(C_0_ − C_PO_)] × 100 (3)

where C_0_ is the initial concentration of benzyl alcohol, and C_PO_ and C_PC_ are the concentrations of PO and PC, respectively, at a certain time after the photocatalytic reaction. The recovered catalyst was separated by centrifugation and washed with methanol three times. After centrifugation and drying under vacuum at 373 K overnight, cyclability experiments were carried out.

## 4. Conclusions

In summary, this work provides an in-depth understanding of visible-light-driven CO_2_ cycloaddition with epoxides over an amino-functionalized UiO-66(Zr) photocatalyst. The unsaturated Zr^4+^ centers act as Lewis acids to facilitate CO_2_ activation, while -NH_2_ groups serve as Lewis basic sites that enhance epoxide adsorption and polarization, thereby collaboratively lowering the activation barriers. As a result, UZN exhibits markedly enhanced photocatalytic performance compared with UZH, achieving an impressive PC yield of 99.5% with near-unit selectivity and a production rate of 9.97 mmol·g^−1^·h^−1^, with a benchmark apparent quantum efficiency of 15.1% at 350 nm. In situ spectroscopy, radical-trapping experiments, and DFT calculations reveal a synergistic mechanism in which CO_2_ activation at Zr^4+^ centers couples with amine-assisted epoxide adsorption, demonstrating a dual-active site-induced catalytic mechanism. This work represents substantial progress in designing efficient photocatalytic platforms based on MOF photocatalysts, offering new insights and methodologies toward high-value-added CO_2_ conversion.

## Figures and Tables

**Table 1:** Substrate scope for conversion of epoxides.

Entry	Substrate	Product	Yield (%)
1	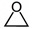	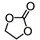	99.8
2	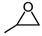	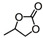	99.5
3	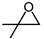	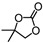	98.2
4	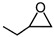	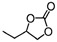	96.0
5	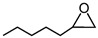	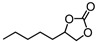	17.3
6	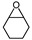	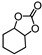	18.4
7	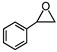	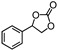	33.8

Reaction conditions: 4 mmol epoxide, 0.04 mmol co-catalyst, solvent CH_3_CN (16 mL), catalyst 40 mg, CO_2_ (1.0 MPa), 300 K, visible light (100 mW·cm^−2^, λ ≥ 420 nm), 10 h. The product yields were quantified by GC-FID with CFCl_3_ as an internal standard.

## Data Availability

The original contributions presented in this study are included in the article/Supplementary Materials. Further inquiries can be directed to the corresponding authors.

## Supplementary Materials

The following supporting information can be downloaded at: https://www.mdpi.com/article/10.3390/molecules31050902/s1. Refs. [21,41,54,55,56,57,58,59,60,61,62,63,64,65,66,67,68,69,70,71] are cited in the Supplementary Materials.
