# Supplementary material for: Boosting Photocatalytic CO2 Cycloaddition via Dual-Active Site Coordination over Amino-Functionalized UiO-66(Zr)
Source: Molecules. 2026 Mar 9;31(5):902. doi: 10.3390/molecules31050902 (PMC12985626; doi:10.3390/molecules31050902)
Supplement: Supplementary file 1 [file molecules-31-00902-s001.zip › molecules-4166186-supplementary.pdf]

Supplementary information

Boosting Photocatalytic CO_2 Cycloaddition via Dual-Active Site Coordination over Amino-Functionalized UiO-66(Zr)

Yajing Lv [1], Haohao Yan [1], Wenhui Ye [1], Lin Ye [2], Jinmei Chen [3,4], Yutong Lin [3,4], Shuying Zhu [1], Dengrong Sun [2,*], Xiyao Liu [3,4,*] and Ruowen Liang [3,4,*]

[1] College of Chemistry, Fuzhou University, Fuzhou 350002, China; lyj010423@163.com (Y.L.); y15038159528@163.com (H.Y.); yyewenhui@163.com (W.Y.); syzhu@fzu.edu.cn (S.Z.)

[2] College of Carbon Neutrality Future Technology, Sichuan University, Chengdu 610064, China; yelin312@scu.edu.cn (L.Y.)

[3] Fujian Provincial Key Laboratory of Featured Biochemical and Chemical Materials, Ningde Normal University, Ningde 352100, China; 15160303767@163.com (J.C.); 13489059799@163.com (Y.L.)

[4] Fujian Province University Key Laboratory of Green Energy and Environment Catalysis, Ningde Normal University, Ningde 352100, China

* Correspondence: dengrongsun@scu.edu.cn (D.S.); t1930@ndnu.edu.cn (X.L.); rwliang@ndnu.edu.cn (R.L.); Tel.: +86-593-2965018 (D.S.); +86-593-2954127 (X.L.); +86-593-296427 (R.L.)

Section S1: Experimental

1.1. Reagents and chemicals

1,4-benzenedicarboxylic acid (H_2BDC) and 2-aminoterephthalic acid (H_2ATA) were obtained from Alfa Aesar China Co., Ltd. (Tianjin, China). $ZrCl_4$ (99.5 wt%), tetrabutylammonium bromide (TBAB), N,N-dimethylformamide (DMF, 99.5 wt%), anhydrous methanol (99.5 wt%), and acetic acid (HAc, 99.5 wt%) were supplied by Sinopharm Chemical Reagent Co., Ltd (Shanghai, China). CO_2 (99.999 %) gas was obtained from Fujian Zhongming High Pressure Gas Co., Ltd (Fuzhou, China).

1.2. Synthesis of UZN and UZH

The UZN particles were synthesized by a traditional solvothermal method. In brief, 2.2 mmol $ZrCl_4$ and 2.0 mmol H_2ATA were dissolved in 45 mL of a mixed solution of HAc and DMF (1:8, v/v). Then, the mixture was stirred for 30 min at 600 rpm, transferred to a 100 mL Teflon liner, and heated at 120 °C for 24 h. UZH was synthesized analogously by replacing H_2ATA with an equivalent molar amount of

H_2BDC. Prior to subjecting the samples to catalytic reactions, an activation process was undertaken. To expunge guest molecules from the pores, the freshly synthesized samples were stirred in anhydrous methanol for 3 d, then filtered and vacuum-dried at 373 K for 12 h.

1.3 Characterization

X-ray diffraction (XRD) patterns were conducted using a D8 Advance X-ray diffractometer (Bruker, Billerica, MA, USA) operated at 40 kV and 40 mA with Ni-filtered Cu Ka irradiation (λ = 0.15406 nm). Scanning electron microscopy (SEM) was performed using an SU8000 scanning electron microscope (Hitachi, Tokyo, Japan). Transmission electron microscopy (TEM) and high-resolution TEM (HRTEM) were performed using a JEM-2010 instrument (JEOL, Tokyo, Japan). The Brunauer–Emmett–Teller (BET) surface area was measured using an ASAP2020M apparatus (Micromeritics, Norcross, GA, USA). Ultraviolet–visible diffuse reflectance spectroscopy (UV-vis DRS) was conducted using a UV-vis spectrophotometer (UV-2700, Shimadzu, Kyoto, Japan). X-ray photoelectron spectroscopy (XPS) was performed using an ESCALAB 250 spectrometer (Thermo Fisher Scientific, Waltham, MA, USA). Photoelectrochemical measurements, including transient photocurrent, Mott–Schottky plots, and electrochemical impedance spectroscopy (EIS), were conducted on an electrochemical workstation (Chenhua CHI-660E) with a standard three-electrode system. Gas chromatography–mass spectrometer (GC-MS) was performed using a Thermo-Fisher Scientific Trace 1300 gas chromatograph equipped with a Thermo-Fischer Scientific ISQ 7000 single-quadrupole mass spectrometer.

1.4 Pyridine-FTIR (Py-FTIR) analysis

Py-FTIR measurements were performed using a Nicolet 6700 to identify Lewis acid sites. The samples were first degassed at 120 ^{o}C for two hours, followed by pyridine adsorption at 25 ^{o}C for one hour. Removal of physisorbed pyridine waas performed at 150 ^{o}C for 30 minutes, after which the spectra were collected.

1.5 Synchrotron X-ray absorption spectra (XAS)

Zr K-edge analysis was performed with Si(111) crystal monochromators at the BL11B beamlines at the Shanghai Synchrotron Radiation Facility (SSRF) (Shanghai, China). Before analysis at the beamline, samples were pressed into thin sheets with a diameter of 1 cm and sealed using Kapton tape film. The XAFS spectra were recorded at room temperature using a four-channel Silicon Drift Detector (SDD) Bruker 5040. Zr K-edge extended X-ray absorption fine structure (EXAFS) spectra were recorded in transmission mode. Negligible changes in the line shape and peak position of Zr K-edge XANES spectra were observed between two scans taken for a specific sample. The XAFS spectra of the standard samples (ZrO_2 and Zr foil) were also recorded in transmission mode. The spectra were processed and analyzed by the software codes Athena and Artemis. Data reduction, data analysis, and EXAFS fitting were performed and analyzed with the Athena and Artemis programs from the Demeter data analysis

packages, which utilizes the FEFF6 program to fit the EXAFS data.[1-3] Energy calibration of the sample was conducted using a standard and Zr foil, which was simultaneously measured as a reference. A linear function was subtracted from the pre-edge region, and the edge jump was normalized using Athena software. The $\chi(k)$ data were isolated by subtracting a smooth third-order polynomial approximating the absorption background of an isolated atom. The k^3-weighted $\chi(k)$ data were Fourier-transformed after applying a HanFeng window function (Δk = 1.0). For EXAFS modeling, the global amplitude EXAFS parameters (CN, R, σ^2 and ΔE_0) were obtained by nonlinear fitting, with least-squares refinement, of the EXAFS equation to the Fourier-transformed data in R-space using Artemis software. The EXAFS of the Zr foil was fitted, and the obtained amplitude reduction factor S_0^2 value (0.958) was set in the EXAFS analysis to determine the coordination numbers (CNs) in the samples.

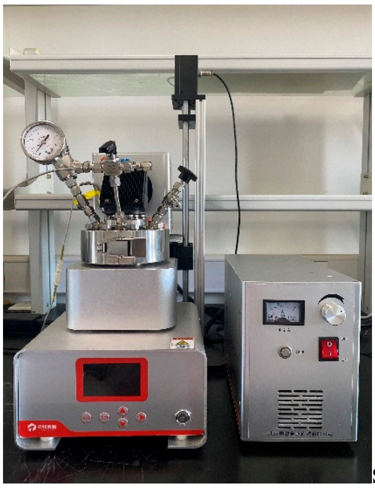
s

Scheme S1. Photograph of the experimental reactor in our lab.

1.6 GC-FID analysis

A Shimazu gas chromatography (GC-2014) with an R-R-smz-C1 autosampler (Shimazu, AOC-20i) was used. Capillary columns (SH-Polar Wax) were used for all injections. Hydrogen was the carrier gas (25 mL/min) and the column temperature control program was as follows: 60 °C initial temperature held for 13.5 min, then 10 °C/min to 170 °C, then 35 °C/min to 250 °C and held for 2.5 min. Detection was performed using an FID (250 °C, hydrogen flow 3.2 mL/min, air flow 25.5 mL/min, nitrogen makeup flow 25 mL/min). Data analysis was performed using OpenLab CDS Chemstation. Calibration curves were generated in the software using weighted linear regression.

1.7 GC-MS analysis

The temperature program for GC-MS analysis was set as follows: initial temperature at 50 °C for 1 min, then raised to 150 °C at 20 °C/min and held for 2 min, and finally raised to 250 ℃ at 10 °C/min and held for 20 min. The split ratio was 1:1, split flow 5.0

mL/min, purge flow 5.0 mL/min, front intel option constant flow 1.5 mL/min, pressure 2 psi. Before injection, we pre-treated the samples as follows: Reaction solution samples collected after the activity test were diluted 1000 times with acetonitrile as the solvent. The sample from the photocatalytic cycloaddition of $^{13}CO_2$ experiment was diluted 100 times, while the samples from the bromine radical capture experiment were injected at their original concentration.

1.8 PC generation under different wavelengths of light

The photocatalytic test was measured using a Xe lamp (Beijing China Education Au-light Co., Ltd., CEL-HXF300-T3) with different band-pass filters (λ = 350±10, 400±10, 450±10, 500±10, 550±10, 600±10, 650±10 and 700±10 nm).

1.9 Determination of apparent quantum yield (AQY)

The AQY of the product for 7%PDI-UZN was measured with irradiation light through different wavelength band-pass filters. The AQY was calculated according to the following equation:[4, 5]

$$AQY = \frac{N_e}{N_p} \times 100\% = \frac{10^9 \times v \times N_A \times K \times h \times c}{I \times A \times \lambda} \times 100\%$$

where N_e stands for the total number of reaction electrons, N_p stands for the number of incident photons, v is the reaction rate (mol·s^{-1}), N_A is the Avogadro constant (6.02×10^{23} mol^{-1}), K represents the number of reaction electrons (in our case, the value of K is 1), h is the Planck constant (6.62×10^{-34} J·s), c is the speed of light (3.0×10^{8} m·s^{-1}), I is the intensity of the irradiation (w·m^{-2}), A stands for the irradiation area (m^2), and λ is the wavelength of incident light (nm).

1.10 Time-resolved in situ diffuse reflectance infrared Fourier transform spectroscopy (In situ FTIR) analysis.

In situ FTIR measurements were performed by employing a Thermo Fisher IS-50 infrared spectrometer equipped with a Harrick diffuse reflectance accessory at the Infrared Spectroscopy and Microspectroscopy Endstation (BL01B) at NSRL in Hefei, China. Firstly, 20 mg of the as-prepared sample was loaded into the cell. A flat gas–solid interface was created and exposed to the reaction atmosphere. Before testing, the entire reaction chamber was purged with Ar (50 mL·min^{-1}) for 30 min at 100 °C to remove all residual impurities. When the chamber temperature was lowered to room temperature, a mixture of 10 mg TBAB, 50 μL of propylene oxide (PO), and CO_2 was injected into the cell. After sorption equilibrium was reached at ~30 min, the background spectrum was acquired, followed by the initial adsorption spectrum (0 min). Thereafter, simulated solar irradiation from a 300 W Xe lamp with a 420 nm cut-off filter was applied, and the in situ FTIR spectra were collected over time to evaluate the intermediate species and the target products under continuous irradiation.

1.11 Computational details

Density functional theory (DFT) calculations on the complete UiO-66 structure were performed using the Vienna Ab initio Simulation Package (VASP) with the projected augmented wave (PAW) method.[6] The exchange–correlation effects were modeled using the revised Perdew–Burke–Ernzerhof (RPBE) functional within the generalized gradient approximation (GGA) [7]. A cut-off energy of 400 eV was used. The catalytic systems were simulated in a cubic cell with dimensions of 20.80 Å per side. For surface calculations, a 1 × 1 × 1 k-point mesh was adopted. Adsorbates were allowed full relaxation during optimization until the convergence criteria were met, with thresholds of 10^{-4} eV for electronic self-consistency and 0.001 eV for structural optimization.

To circumvent artifacts from coordinate-interpolation approaches, transition states were located by direct searches in Gaussian 16 [8]. Spin-polarized DFT calculations employed the B3LYP hybrid GGA functional with Grimme's DFT-D3 dispersion correction. All geometries were fully optimized and energies evaluated with the def2-SVP basis set for all elements. Each transition state was validated by vibrational analysis, exhibiting a single imaginary frequency. Independent Gradient Model based on Hirshfeld partitioning (IGMH) [9] analysis was performed using Multiwfn, [10] and the results were visualized with VMD.[11]

In the context of molecular adsorption, the adsorption energy ($\boldsymbol{E}_{ad}$) of species is A defined as follows: $\boldsymbol{E}_{ad}(A) = \boldsymbol{E}_{A+M} - \boldsymbol{E}_{M} - \boldsymbol{E}_{A}$, where $\boldsymbol{E}_{A+M}$, $\boldsymbol{E}_{M}$, and $\boldsymbol{E}_{A}$ denote the total energies of the catalyst with adsorbed A, the pristine catalyst, and the isolated molecule A, respectively. For the computed free-energy profiles, the activation barrier $\boldsymbol{E}_{a}$ is defined as $\boldsymbol{E}_{a} = \boldsymbol{E}_{TS} - \boldsymbol{E}_{IS}$, where $\boldsymbol{E}_{IS}$ and $\boldsymbol{E}_{TS}$ denote the initial and transition states, respectively.

Section S2: Characterizations

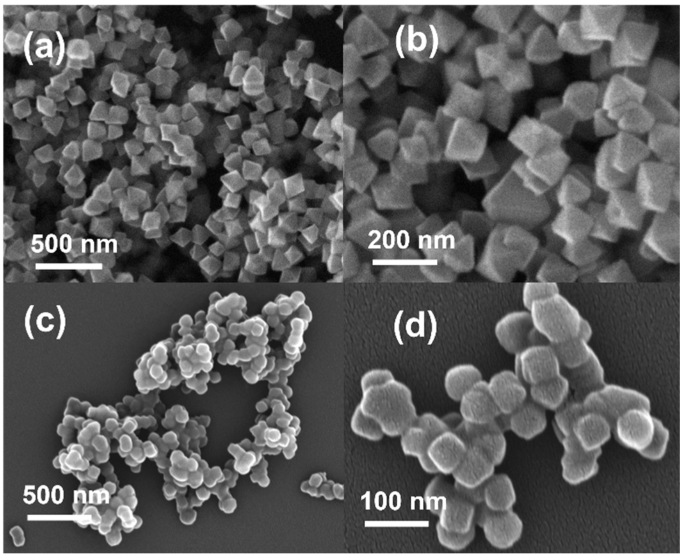

Figure S1. SEM images of (a)-(b) UZN and (c)-(d) UZH.

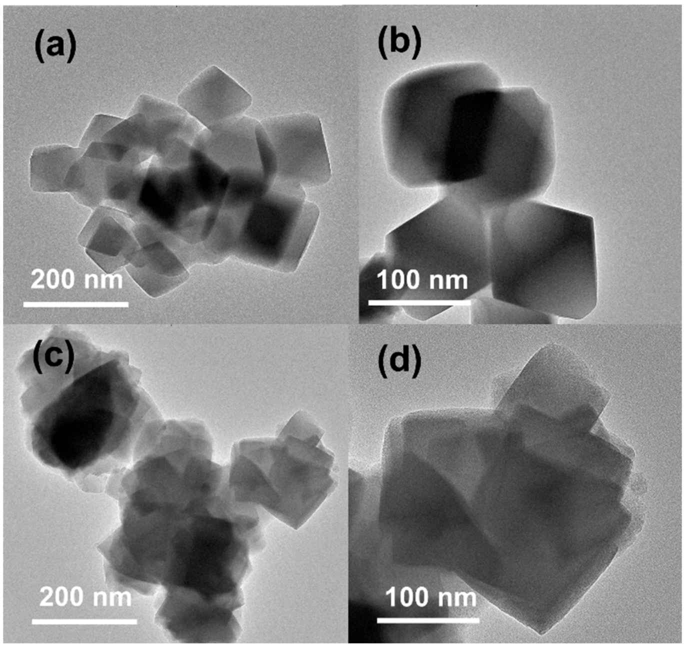

Figure S2. TEM images of (a)-(b) UZN and (c)-(d) UZH.

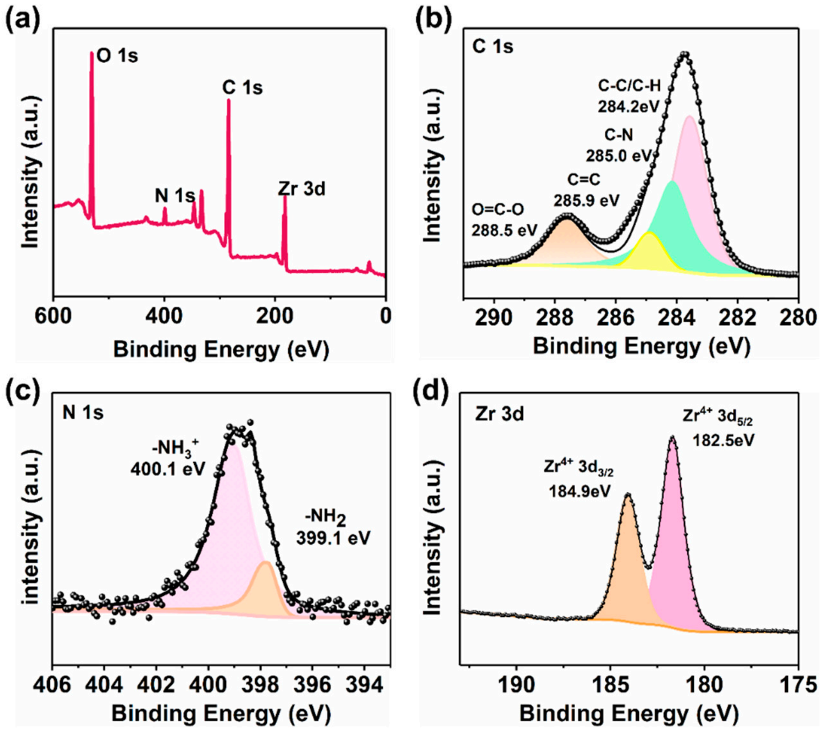

Figure S3. (a) XPS survey spectrum of UZN. High-resolution XPS spectra of (b) C 1s, (c) N 1s and (d) Zr 3d for UZN.

Table S1. EXAFS fitting parameters at the Zr K-edge for various samples

Sample	*Shell*	*CN*[a]	*R(Å)*[b]	$\sigma^2(Å^2)^c$	$\Delta E_0(eV)^d$	*R factor*
Zr foil	Zr-Zr	12*	3.21±0.01	0.0094	4.1	0.0069
ZrO_2	Zr-O	7.8±1.3	2.16±0.01	0.0117	-5.4	0.0077
	Zr-Zr	8.6±2.2	3.46±0.01	0.0088	-9.4	
UZN	Zr-O	2.6±1.0	2.07±0.01	0.0052	-10.0	0.0077
	Zr-O	4.0±0.5	2.30±0.01	0.0066	9.8	
	Zr-Zr	2.8±0.5	3.52±0.01	0.0035	-3.1	

[a]*CN*, coordination number; [b]*R*, distance between absorber and backscatter atoms; [c]σ^2, Debye–Waller factor to account for both thermal and structural disorders; [d]ΔE_0, inner potential correction; *R* factor indicates the goodness of fit. S_0^2 was fixed to 0.958 according to the experimental EXAFS fit of Zr foil by fixing CN as the known crystallographic value. A reasonable range of EXAFS fitting parameters is as follows: $0.600 < S_0^2 < 1.000$; $CN > 0$; $\sigma^2 > 0$ Å^2; $|\Delta E_0| < 15$ eV; *R* factor < 0.02.

Table S2 Number of Lewis acidic sites (μmol·g^{-1}) from in situ FTIR results of pyridine adsorption analysis.

Sample	*Lewis acid sites (μmol·g^{-1})*
UZN	152.3
UZH	152.2
U-UZN	120.2

Table S3. Photocatalytic cycloaddition of CO_2 with PO to synthesize PC[a].

Entry	*Cat./Co-Cat.*	*Yield (%)*	*Conversion (%)*	*Selectivity (%)*	*Reaction Rate (mmol·g^{-1}·h^{-1})*
1	None	1.8	1.8	100.0	0.18
2	None/TBAB	4.4	4.4	100.0	0.45
3	UZN/TBAB/Dark	5.9	6.0	98.3	0.59
4	UZN/None	3.5	3.5	100.0	0.35
5	UZH/TBAB	7.9	8.0	98.8	0.77
6	UZN/TBAB	99.5	99.7	99.7	9.97
7	U-UZN/TBAB	54.1	56.5	95.8	5.26

[a] Reaction conditions: 4 mmol PO, 0.04 mmol TBAB, solvent CH_3CN (16 mL), CO_2 (1.0 MPa), 300 K, catalyst 40 mg, visible light (100 mW·cm^{-2}, $\lambda \geq 420$ nm), 10 h. The product yields were quantified by GC-FID with $CFCl_3$ as an internal standard.

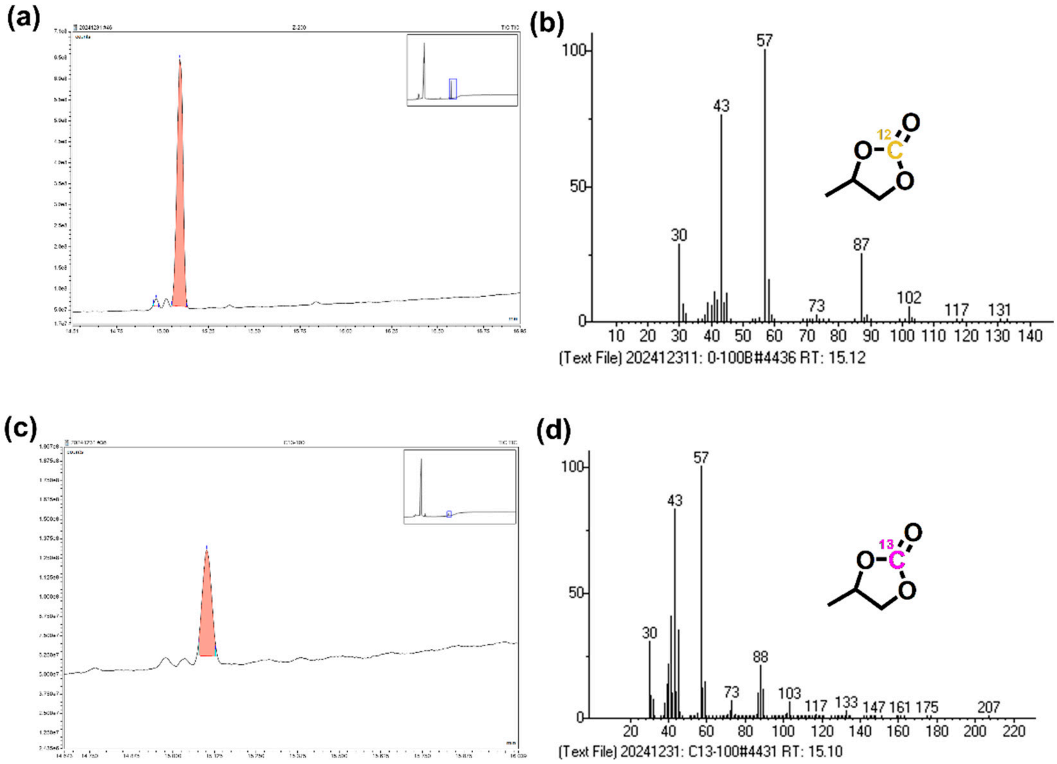

Figure S4. GC-MS spectrum of the target product generated by photocatalytic cycloaddition of $^{13}CO_2$ and PO.

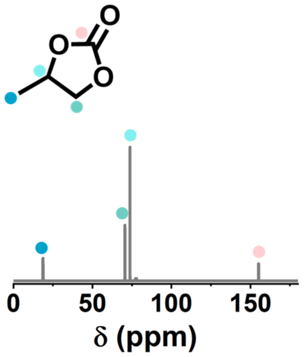

Figure S5. ^{13}C NMR of PC product.

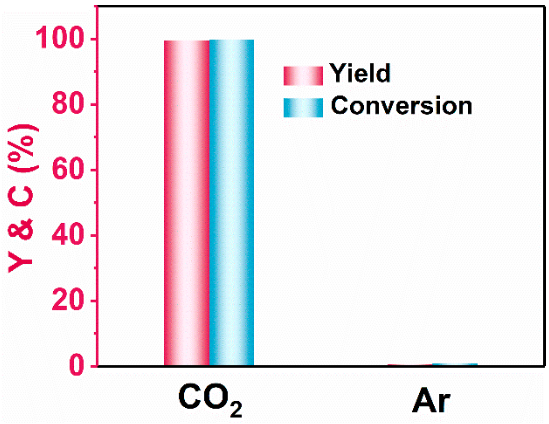

Figure S6. Comparison of the catalytic performance of UZN under different reaction atmosphere conditions.

Table S4. Photocatalytic cycloaddition of CO_2 with PO to synthesize PC using different co-catalysts[a]

Entry	*Cat./Co-Cat.*	*Yield (%)*	*Conversion (%)*	*Selectivity (%)*	*Reaction rate (mmol·g⁻¹·h⁻¹)*
1	UZN/TBAB	99.5	99.8	99.7	9.97
2	UZN/TBAI	80.1	99.7	80.3	7.97
3	UZN/TBAF	71.4	93.9	76.0	7.18
4	UZN/$ZnBr_2$	64.6	99.2	65.1	6.62

[a] Reaction conditions: 4 mmol PO, 0.04 mmol co-catalyst, solvent CH_3CN (16 mL), CO_2 (1.0 MPa), 300 K, catalyst 40 mg, visible light (100 mW·cm^{-2}, $\lambda \geq 420$ nm), 10 h. The product yields were quantified by GC-FID with $CFCl_3$ as an internal standard.

Table S5. Photocatalytic cycloaddition of CO_2 over UZN under various conditions[a].

Entry	*UZN (mg)*	*TB[illegible]B (mmol)*	*Time (h)*	*P (MPa)*	*Yield (%)*	*Conversion (%)*	*Selectivity (%)*	*Reaction rate (mmol·g⁻¹·h⁻¹)*
1	40	0.04	10	1.0	99.5	99.8	99.7	9.97
2	20	0.04	10	1.0	48.4	48.4	99.6	9.39
3	30	0.04	10	1.0	74.6	74.6	99.7	9.90
4	50	0.04	10	1.0	99.5	99.7	99.8	7.86
5	60	0.04	10	1.0	99.6	99.8	99.8	6.54
6	40	0.02	10	1.0	54.8	55.9	98.0	5.32

7	40	0.03	10	1.0	82.8	83.2	99.5	8.24
8	40	0.05	10	1.0	98.2	98.8	99.4	9.70
9	40	0.06	10	1.0	99.1	99.3	99.8	9.76
10	40	0.04	5	1.0	59.2	59.7	99.2	12.04
11	40	0.04	7.5	1.0	80.2	81	99.0	11.01
12	40	0.04	12.5	1.0	99.3	99.4	99.9	7.98
13	40	0.04	15	1.0	99.8	100.0	99.8	6.74
14	40	0.04	10	0.1	32.8	34.4	95.3	3.19
15	40	0.04	10	0.5	78.2	79.7	98.1	8.04
16	40	0.04	10	1.5	99.2	99.6	99.6	9.62
17	40	0.04	10	1.8	99.0	99.4	99.6	9.68

[a] Reaction conditions: Solvent CH_3CN (16 mL), CO_2 (1.0 MPa), 300 K, visible light (100 $mW \cdot cm^{-2}$, $\lambda \geq 420$ nm). The product yields were quantified by GC-FID with CF_3Cl as an internal standard.

Table S6. Photocatalytic cycloaddition of CO_2 over UZN under different light wavelengths[a].

Entry	*Light wavelength (nm)*	*Yield (%)*	*Conversion (%)*	*Selectivity (%)*	*Reaction rate ($mmol \cdot g^{-1} \cdot h^{-1}$)*
1	254	31.5	57.3	99.8	5.6
2	310	31.0	57.0	99.3	5.7
3	350	8.2	34.1	99.4	3.4
4	400	54.3	81.2	98.4	7.6
5	420	76.9	99.3	99.6	9.6
6	450	18.3	44.4	98.9	4.3
7	500	20.6	47.5	97.3	4.6
8	600	10.6	37.3	97.0	3.6
9	700	14.3	40.9	97.6	4.1

[a] Reaction conditions: 4 mmol PO, 0.04 mmol co-catalyst, solvent CH_3CN (16 mL), CO_2 (1.0 MPa), 300 K, catalyst 40 mg, 10 h. The product yields were quantified by GC-FID with $CFCl_3$ as an internal standard.

Table S7. AQY of the product under different monochromatic light conditions.

Entry	*Light wavelength (nm)*	*Intensity of irradiation ($mW \cdot cm^{-2}$)*	*Yield (%)*	*□QY (100%)*
1	254	6.4	31.5	16.7
2	310	6.4	31.0	13.4
3	350	1.4	8.2	15.1
4	400	10.7	54.3	11.3

5	420	20.2	76.9	7.2
6	450	10.8	18.3	3.2
7	500	11.8	20.6	3.0
8	600	27.9	10.6	0.6
9	700	17.5	14.3	1.0

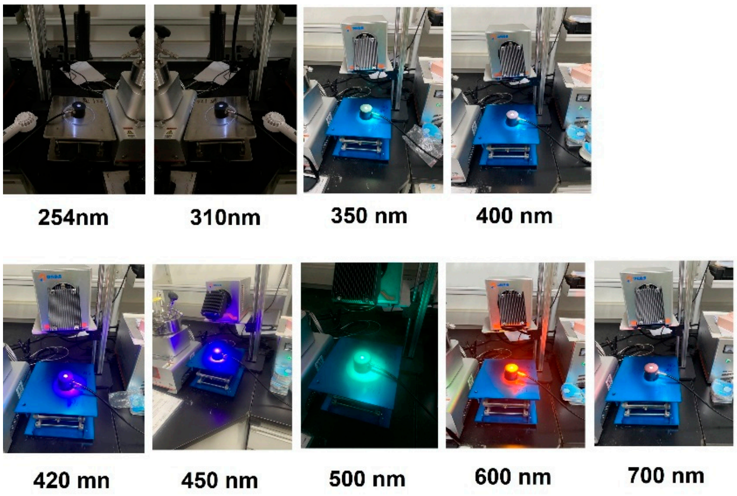

Figure S7. Photograph of the light intensity test experiment in our lab.

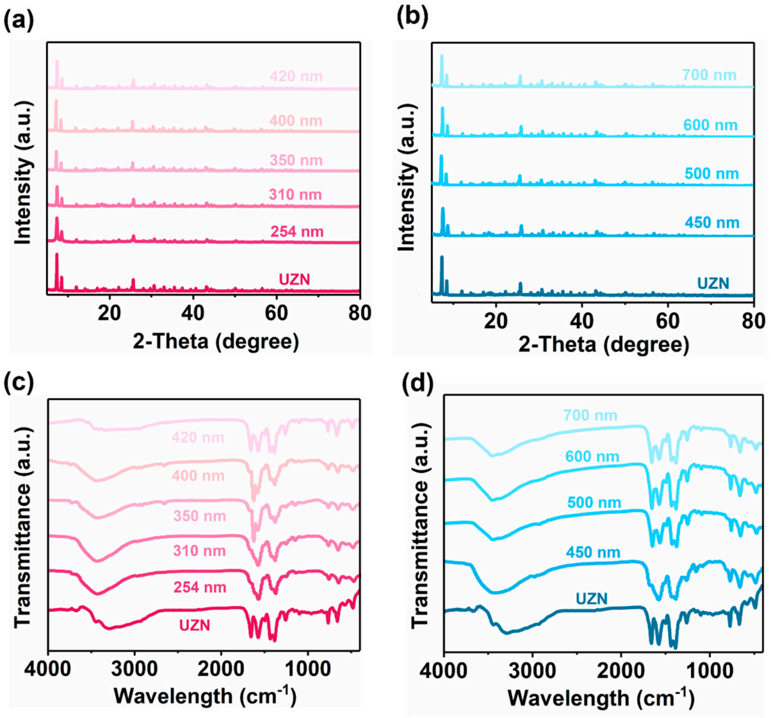

Figure S8. (a)-(b) XRD of recovered UiO-66-NH_2 after reaction, (c)-(d) XRD of recovered UiO-66-NH_2 after reaction.

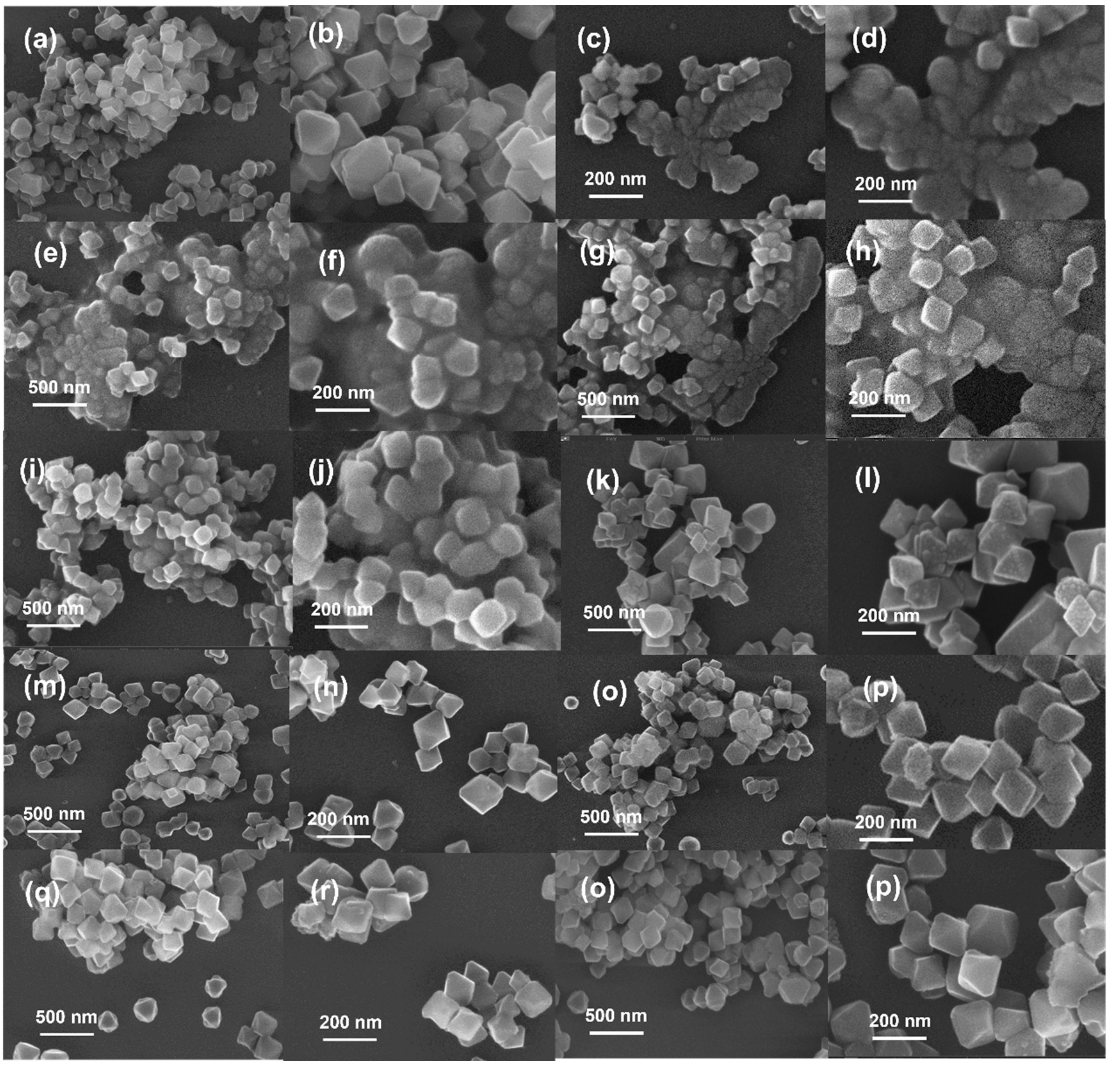

Figure S9. SEM images of the recovered sample (a)-(b) before reaction, (c)-(d) 254 nm, (e)-(f) 310 nm, (g)-(h) 350 nm, (i)-(j) 400 nm, (k)-(l) 420 mn, (m)-(n) 450 nm, (o)-(p) 500 nm, (q)-(r) 600 nm, (s)-(t) 700 nm.

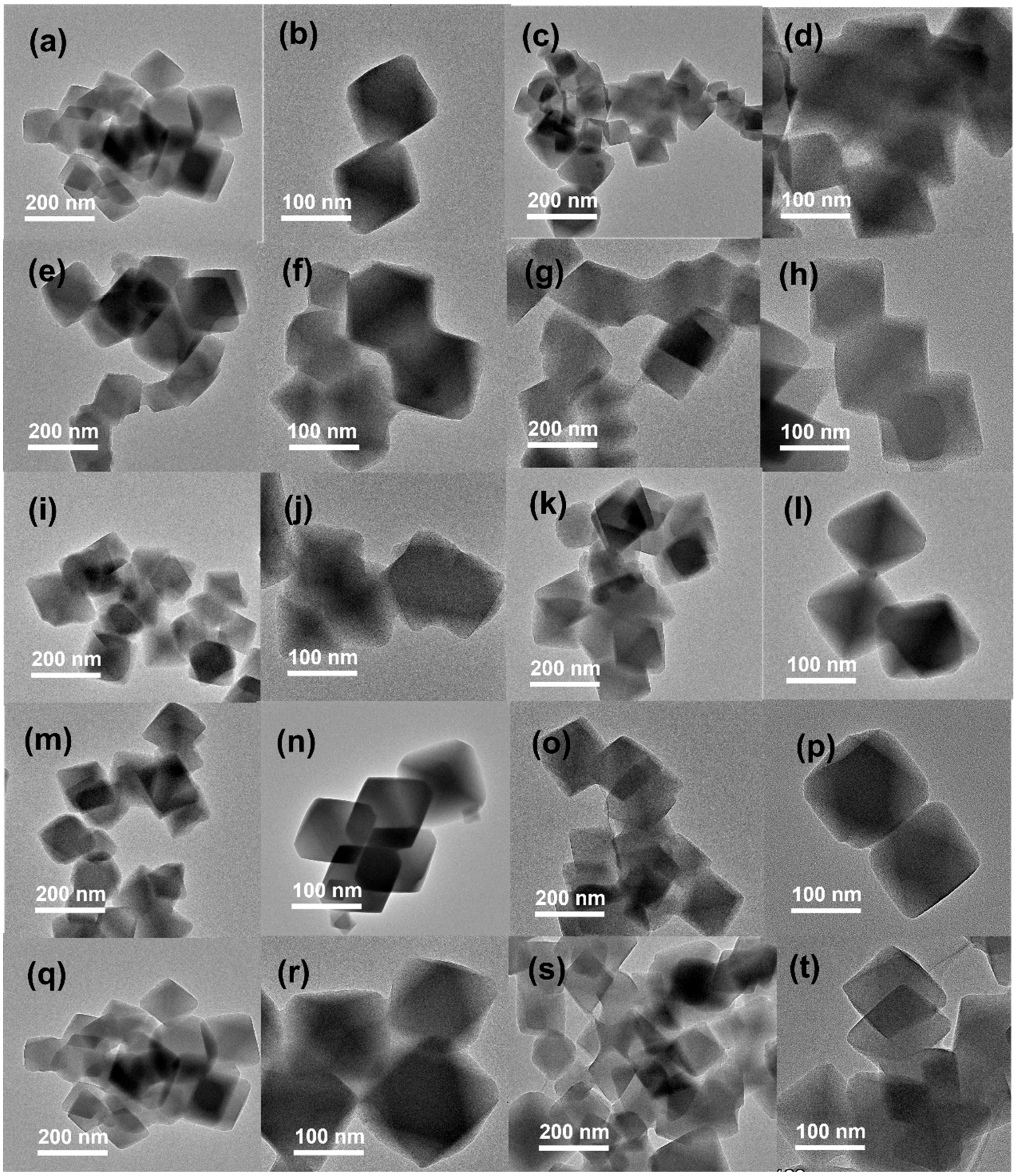

Figure. S10 TEM images of the recovered sample (a)-(b) before reaction , (c)-(d) 254 nm, (e)-(f) 310 nm, (g)-(h) 350 nm, (i)-(j) 400 nm, (k)-(l) 420 mn, (m)-(n) 450 nm, (o)-(p) 500 nm, (q)-(r) 600 nm, (s)-(t) 700 nm.

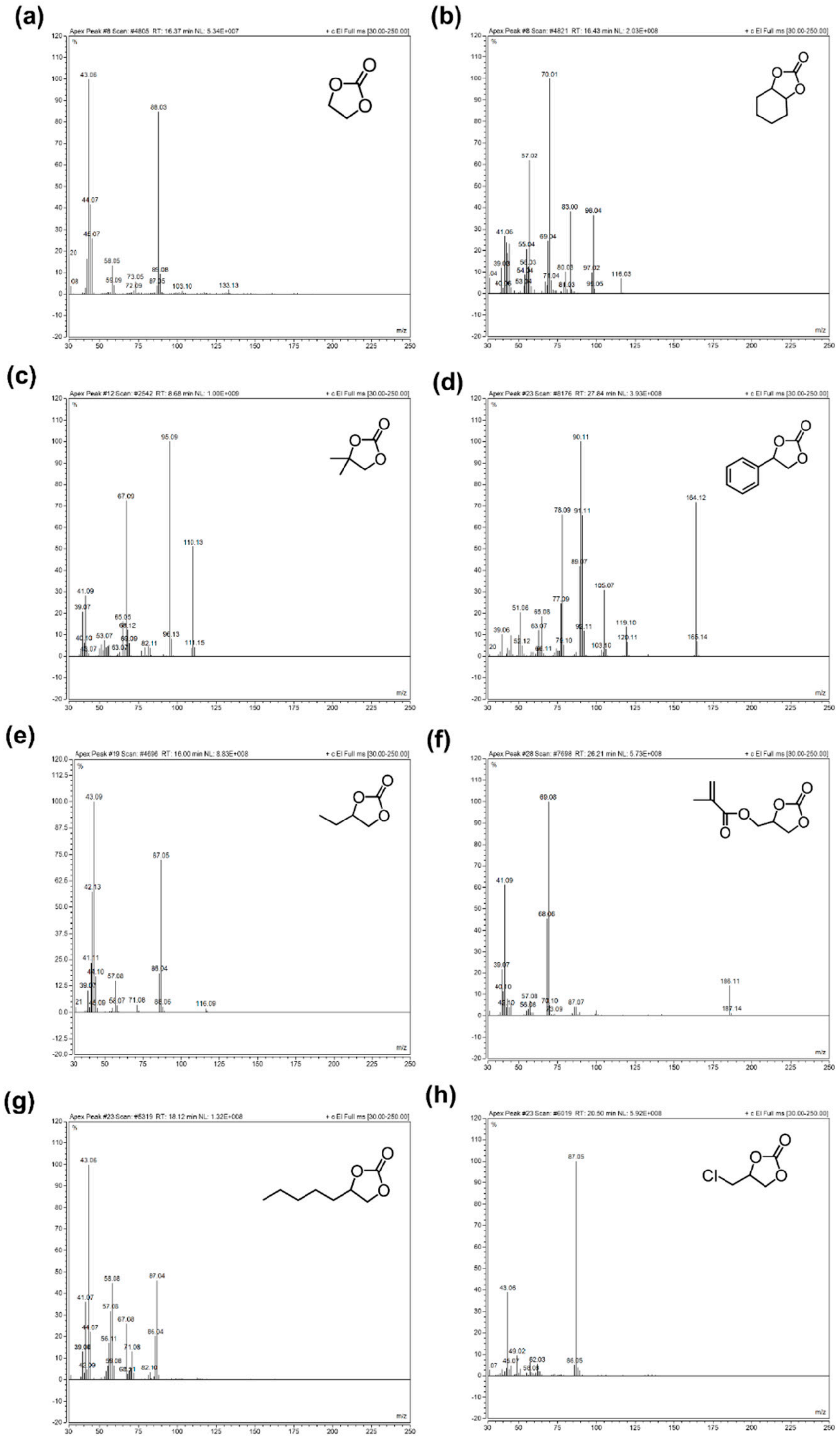

Figure S11. GC analyses of the obtained products after photocatalysis.

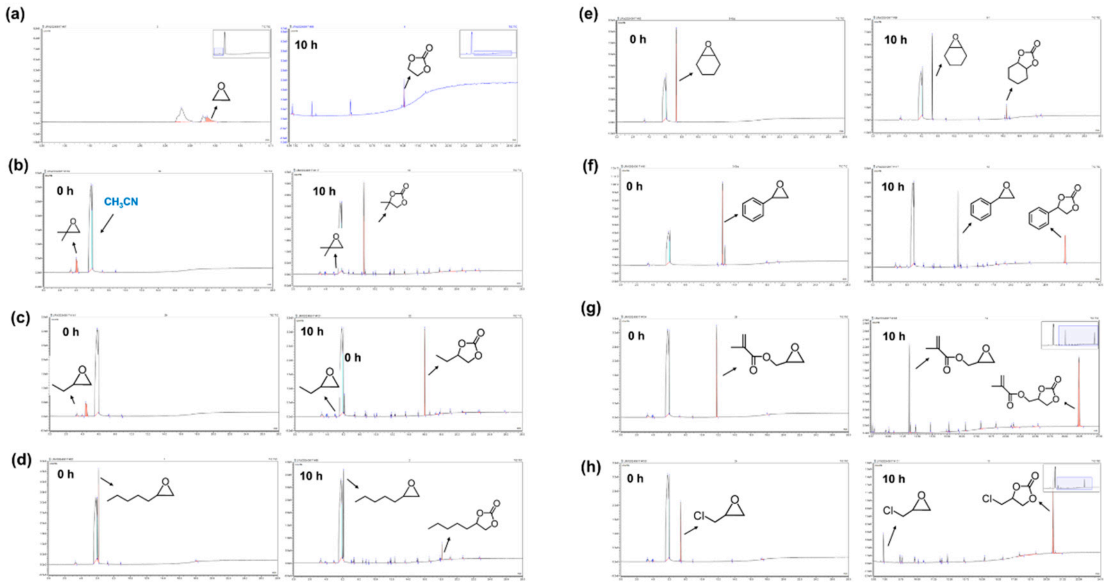

Figure S12. GC-MS analyses of the obtained products.

Table S8. Comparison of recent studies on photocatalytic CO_2 cycloaddition systems.

Entry	*Catalyst*	*Co-catalyst*	*Temperature*	*Reaction time (h)*	*CO_2 Pressure*	*Light source*	*Conversion (%)*	*Yield (%)*	*Selectivity (%)*		*Ref.*
1	UZN	TBAB	300 K	10	1 MPa	100 mW·cm^{-2} visible light ($\lambda \geq 420$ nm)	99.8	99.5	99.7	9.97	This work
2	Co-^{s}ZIS	TBAB	298 K	4	1 bar	300 mW·cm^{-2} visible light ($\lambda > 420$ nm)	60	57	95	7.21	12
3	$Ti_{18}Bi_4O_{29}Bz_{26}$	TBAB	298 K	12	1 bar	300 W xenon lamp	40	38	95	2.12	13
4	$BiNbO_4$/r-GO	TBAB	353 K	6	1.48 MPa	300 W halogen lamp	5	5	100	0.0054	14
5	UiO-67-B	TBAB	298 K	12	1 bar	Xenon lamp (300 W 18 A)	37.2	37.2	99	3.72	15
6	Zn-SA-NC	TBAB	298 K	10	1 bar	300 mW·cm^{-2} full-spectrum	12	11	92	0.45	16
7	$FeNbO_4$/ NH_2-MIL125(Ti)	TBAB	348 K	12	0.045 mol CO_2	500 W visible light halogen lamp	20	20	100	0.2	17
8	Ce-BDC-NH_2	TBAB	298 K	12	1 bar	125 W medium-pressure mercury vapor lamp	55	52	94.5	0.12	18
9	$BiNbO_4$/ NH_2-MIL125(Ti)	TBAB	353 K	6	0.045 mol CO_2	300 W halogen lamp	46	45	97.8	0.062	19
10	IL-UiO-66-NH_2	None	373K	8	1 MPa	None	99	94	95	5.88	20
11	Defective NH_2-UiO-66(Zr)	None	363K	10	0.1 MPa	None	98	93	95	4.65	21
12	IL-UiO-66	None	373K	6	1 MPa	None	97	90	93	7.50	22

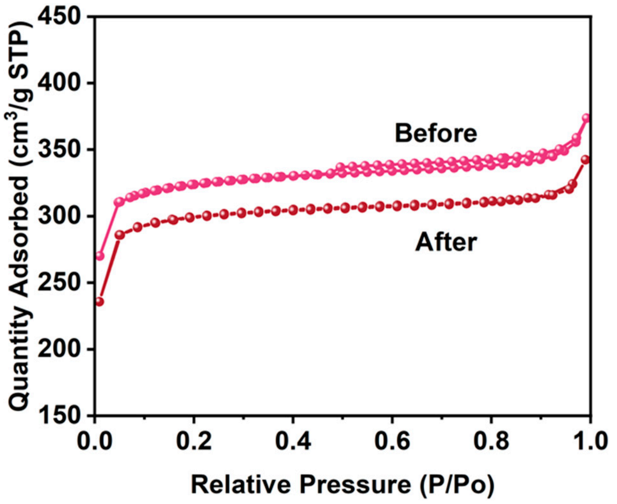

Figure S13. N_2 adsorption–desorption isotherm of UZN before and after the reaction.

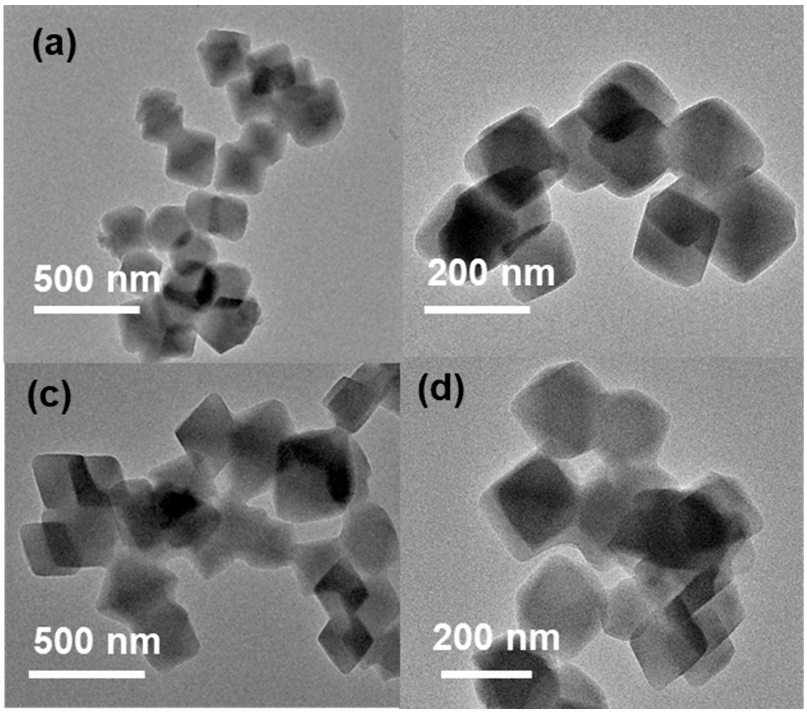

Figure S14. TEM images of UZN: (a)-(b) before and (c)-(d) after the reaction.

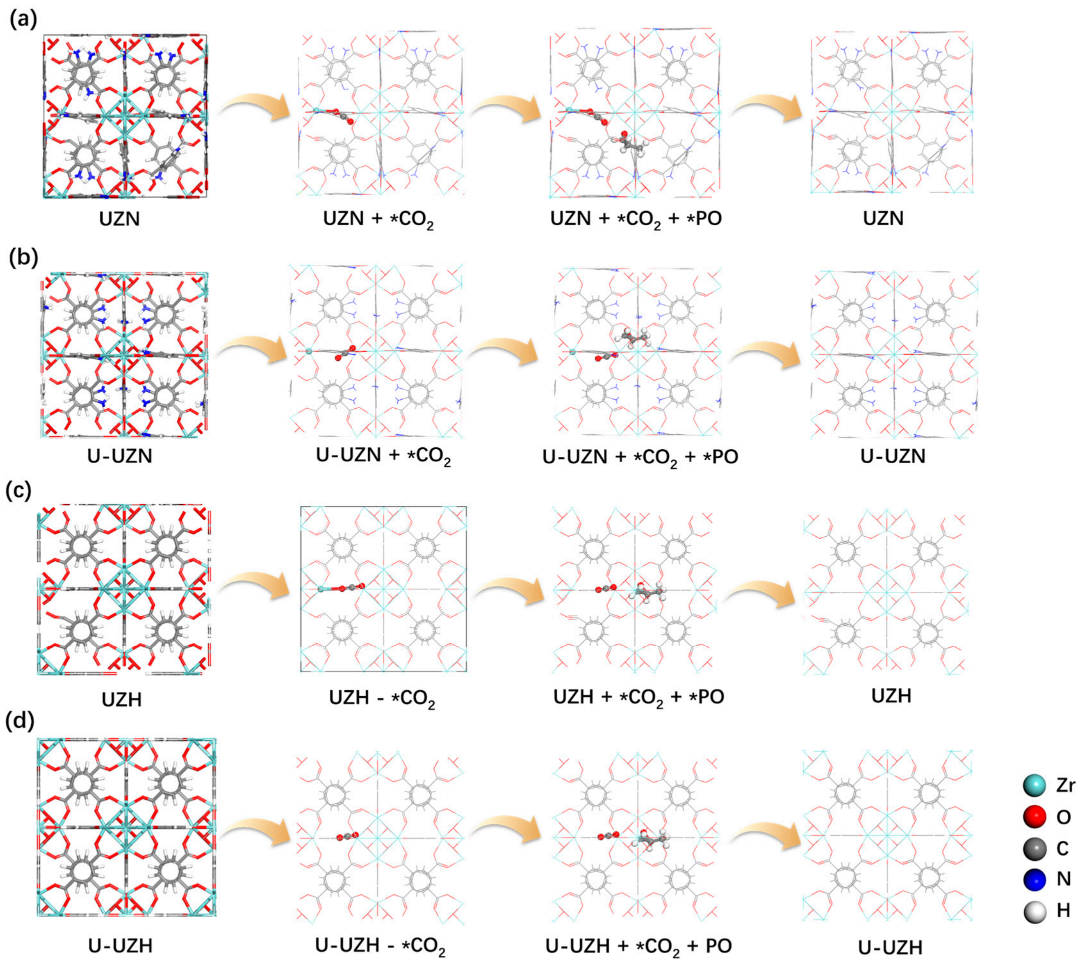

Figure S15. Structural images of each intermediate along the adsorption pathway.

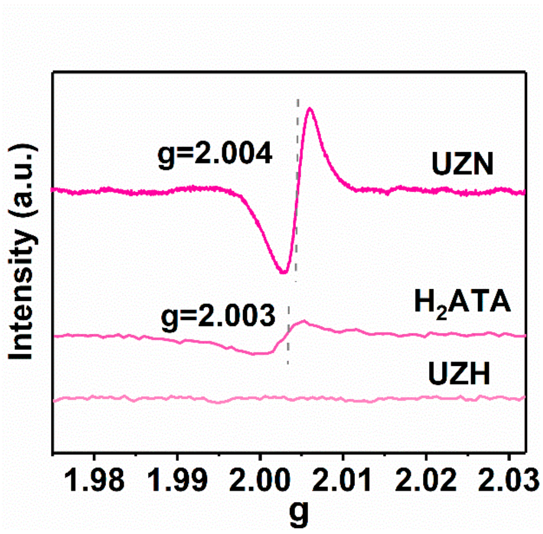

Figure S16. ESR spectra of UZN, UZH, and H_2ATA under visible-light irradiation.

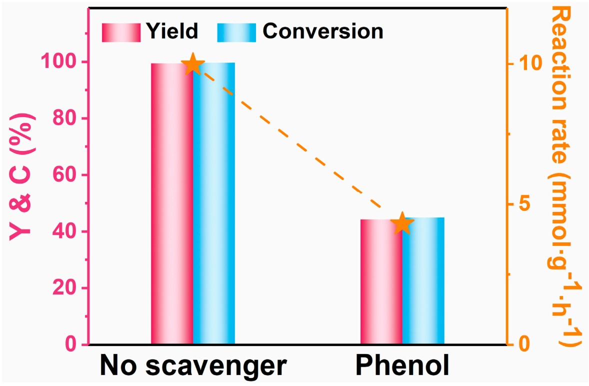

Figure S17. Photocatalytic synthesis of PC in the presence of phenol (a •Br scavenger, 0.1 mmol).

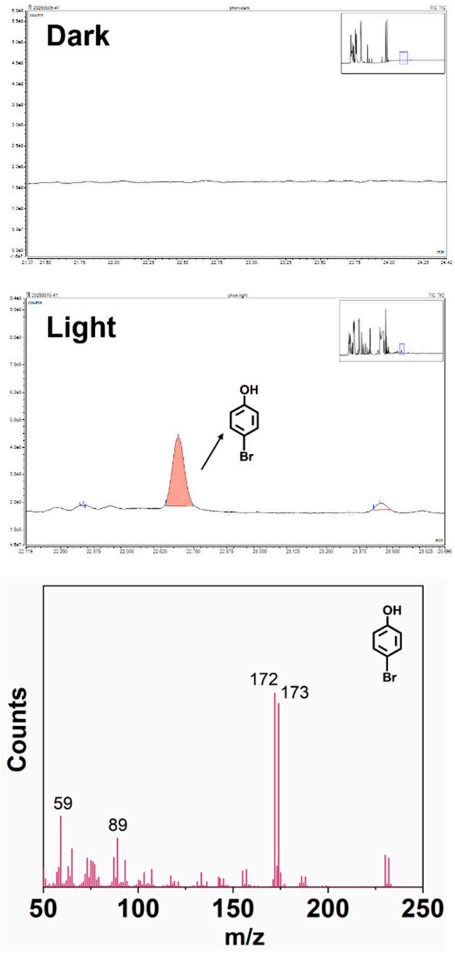

Figure S18. GC-MS spectrum of the experimental products obtained from the •Br trapping experiment over UZN.

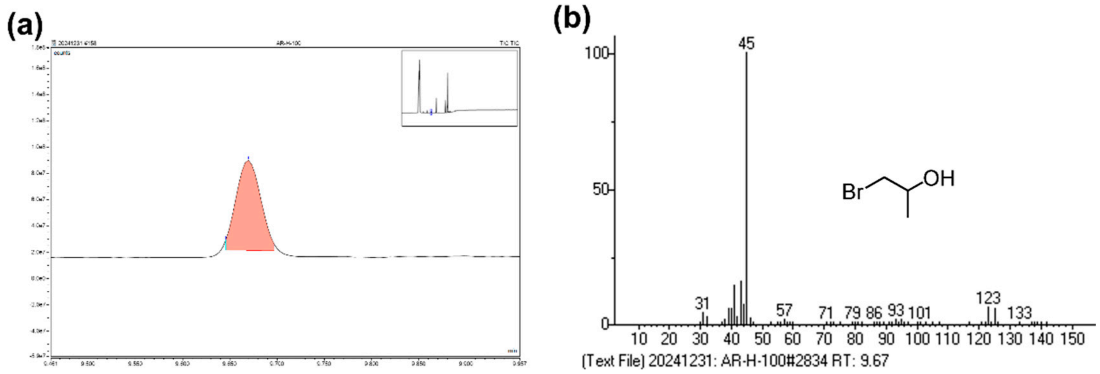

Figure S19. GC-MS spectrum of PO ring-opened intermediate (C_3H_6OBr) generated by •Br attacking over UZN.

References

1. Lomachenko, K.; Jacobsen, J.; Bugaev, A.; Atzori, C.; Bonino, F.; Bordiga, S.; Stock, N.; Lamberti, Carlo., Exact Stoichiometry of Ce_xZr_{6-x} Cornerstones in Mixed-Metal UiO-66 Metal-organic Frameworks Revealed by Extended X-ray Absorption Fine Structure Spectroscopy. *Journal of the American Chemical Society*, **2018**, 140, (50), 17379-17383. https://doi.org/10.1021/jacs.8b10343

2. Finzel, J.; Gutierrez, K.; Hoffman, A.; Resasco, J.; Christopher, P.; Bare, S., Limits of Detection for EXAFS Characterization of Heterogeneous Single-atom Catalysts. *ACS Catalysis*, **2023**,13, (9), 6462-6473. https://doi.org/10.1021/acscatal.3c01116

3. Su, Z.; Miao, Y.; Zhang, G.; Miller, J.; Suslick, K., Bond Breakage under Pressure in a Metal Organic Framework. *Chemical Science*, **2017**, 8, (12), 8004-8011. https://doi.org/10.1039/C7SC03786D

4. Ricka, R.; Přibyl, M.; Kočí, K., Apparent Quantum Yield-Key Role of Spatial Distribution of Irradiation. *Applied Catalysis A: General*, **2023**, 658, 119166. https://doi.org/10.1016/j.apcata.2023.119166

5. Musa, E.; Yadav, A.; Smith, K.; Jung, M.; Stickle, W.; Eschbach, P.; Ji, X.; Stylianou, K., Boosting Photocatalytic Hydrogen Production by MOF-Derived Metal Oxide Heterojunctions with a 10.0% Apparent Quantum Yield. *Angewandte Chemie-International Edition*, **2024**, 63, (42), e202405681. https://doi.org/10.1002/anie.202405681

6. Kresse, G.; Hafner, J., Ab Initio Molecular Dynamics for Liquid Metals. *Physical Review B*, **1993**, 47, (1), 558-561. https://doi.org/10.1103/PhysRevB.47.558

7. Blöchl, P. E., Projector Augmented-Wave Method. *Physical Review B*, **1994**, 50, (24), 17953–17979. https://doi.org/10.1103/PhysRevB.50.17953

8. Frisch, M. J.; Trucks, G. W.; Schlegel, H. B.; Scuseria, G. E.; Robb, M. A.; Cheeseman, J. R.; Scalmani, G.; Barone, V.; Petersson, G. A.; Nakatsuji, H.; et al., Gaussian 16, Revision A.03. Gaussian, Inc., *Wallingford, CT*, 2016.

9. Lu, T.; Chen, Q. X., Independent Gradient Model Based on Hirshfeld Partition: A New Method for Visual Study of Interactions in Chemical Systems. *Journal of Computational Chemistry*, **2022**, 43, (8), 539-555. https://doi.org/10.1002/jcc.26812.

10. Lu, T.; Chen, F. W., Multiwfn: A Multifunctional Wavefunction Analyzer. *Journal of Computational Chemistry*, **2012**, *33*, (5), 580-592. https://doi.org/10.1002/jcc.22885.

11. Humphrey, W.; Dalke, A.; Schulten, K., VMD: Visual Molecular Dynamics. *Journal of Molecular Graphics*, **1996**, *14*, (1), 33-38. https://doi.org/10.1016/0263-7855(96)00018-5.

12. Tan, C.; Qi, M.; Tang, Z.; Xu, Y., Isolated Single-Atom Cobalt in the $ZnIn_2S_4$ Monolayer with Exposed Zn Sites for CO_2 Photofixation. *ACS Catalysis*, **2023**, 13, (12), 8317-8329. https://doi.org/10.1021/acscatal.3c00992

13. Liu, C.; Niu, H.; Wang, D.; Gao, C.; Said, A.; Liu, Y.; Wang, G.; Tung, C.; Wang, Y., S-Scheme Bi-oxide/Ti-oxide Molecular Hybrid for Photocatalytic Cycloaddition of Carbon Dioxide to Epoxides. *ACS catalysis*, **2022**,14, (12), 8202-8213. https://doi.org/10.1021/acscatal.2c02256

14. Bakiro, M.; Ahmed, S.; Alzamly, A., Efficient Visible-Light Photocatalytic Cycloaddition of CO_2 and Propylene

Oxide Using Reduced Graphene Oxide Supported $BiNbO_4$. *ACS Sustainable Chemistry & Engineering,* **2020**, 8, (32), 12072-12079.
https://doi.org/10.1021/acssuschemeng.0c03363
15. Li, Y.; Zhai, G.; Liu, Y.; Wang, Z.; Wang, P.; Zheng, Z.; Cheng, H.; Dai, Y.; Huang, B., Synergistic Effect Between Boron Containing Metal-organic Frameworks and Light Leading to Enhanced CO_2 Cycloaddition with Epoxides. *Chemical Engineering Journal,* **2022**, 437, 135363.
https://doi.org/10.1016/j.cej.2022.135363
16. Gong, L.; Sun, J.; Liu, Y.; Yang, G., Photoinduced Synergistic Catalysis on Zn Single-atom-loaded Hierarchical Porous Carbon for Highly Efficient CO_2 Cycloaddition Conversion. *Journal of Materials Chemistry A*, **2021**, 9, (38), 21689-21694.
https://doi.org/10.1039/D1TA06159C
17. Ahmed, S.; Bakiro, M.; Alzamly, A., Photocatalytic Activities of $FeNbO_4$/NH_2-MIL-125(Ti) Composites toward the Cycloaddition of CO_2 to Propylene Oxide. *Molecules,* **2021**, 26, (6), 1693.
https://doi.org/10.3390/molecules26061693
18. Payra, S.; Roy, S., From Trash to Treasure: Probing Cycloaddition and Photocatalytic Reduction of CO_2 over Cerium-Based Metal-organic Frameworks. *The journal of physical chemistry, C. Nanomaterials and interfaces*, **2021**, 125, (16), 8497-8507.
https://doi.org/10.1021/acs.jpcc.1c00662
19. Bakiro, M.; Ahmed, S.; Ahmed, A., Cycloaddition of CO_2 to Propylene Oxide using $BiNbO_4$/NH_2-MIL-125(Ti) Composites as Visible-light Photocatalysts. *Journal of Environmental Chemical Engineering,* **2020**, 8, (6), 104461.
https://doi.org/10.1016/j.jece.2020.104461
20. Wu, Y.; Xiao, Y.; Yuan, H.; Zhang, Z.; Shi, S.; Wei, R.; Gao, L.; Xiao, G., Imidazolium Ionic Liquid Functionalized UiO-66-NH_2 as Highly Efficient Catalysts for Chemical Fixation of CO_2 into Cyclic Carbonates. *Microporous Mesoporous Mater.*, **2021**, 310, 110578.
https://doi.org/10.1016/j.micromeso.2020.110578
21. Liu, X.; Hu, C.; Wu, J.; Cui, P.; Wei, F., Defective NH_2-UiO-66 (Zr) Effectively Converting CO_2 into Cyclic Carbonate under Ambient Pressure, Solvent-Free and Co-Catalyst-Free Conditions. *Chinese Journal of Chemical Engineering,* **2022**, 43, 222-229.
https://doi.org/10.1016/j.cjche.2022.02.016
22. Kurisingal, J.; Rachuri, Y.; Pillai, R.; Gu, Y.; Choe, Y.; Park, D., Ionic-Liquid-Functionalized UiO-66 Framework: An Experimental and Theoretical Study on the Cycloaddition of CO_2 and Epoxides. *ChemSusChem*, **2019**, 12, 1033-1042.
https://doi.org/10.1002/cssc.201802838
